# Impact of data processing varieties on DCM estimates of effective connectivity from task‐fMRI

**DOI:** 10.1002/hbm.26751

**Published:** 2024-06-12

**Authors:** Shufei Zhang, Kyesam Jung, Robert Langner, Esther Florin, Simon B. Eickhoff, Oleksandr V. Popovych

**Affiliations:** ^1^ Institute of Neuroscience and Medicine, Brain and Behaviour (INM‐7) Research Centre Jülich Jülich Germany; ^2^ Institute for Systems Neuroscience, Medical Faculty Heinrich‐Heine University Düsseldorf Düsseldorf Germany; ^3^ Institute of Clinical Neuroscience and Medical Psychology, Medical Faculty Heinrich‐Heine University Düsseldorf Düsseldorf Germany

**Keywords:** analytical flexibility, global signal regression, MRI data processing, stimulus–response compatibility, task‐evoked effective connectivity

## Abstract

Effective connectivity (EC) refers to directional or causal influences between interacting neuronal populations or brain regions and can be estimated from functional magnetic resonance imaging (fMRI) data via dynamic causal modeling (DCM). In contrast to functional connectivity, the impact of data processing varieties on DCM estimates of task‐evoked EC has hardly ever been addressed. We therefore investigated how task‐evoked EC is affected by choices made for data processing. In particular, we considered the impact of global signal regression (GSR), block/event‐related design of the general linear model (GLM) used for the first‐level task‐evoked fMRI analysis, type of activation contrast, and significance thresholding approach. Using DCM, we estimated individual and group‐averaged task‐evoked EC within a brain network related to spatial conflict processing for all the parameters considered and compared the differences in task‐evoked EC between any two data processing conditions via between‐group parametric empirical Bayes (PEB) analysis and Bayesian data comparison (BDC). We observed strongly varying patterns of the group‐averaged EC depending on the data processing choices. In particular, task‐evoked EC and parameter certainty were strongly impacted by GLM design and type of activation contrast as revealed by PEB and BDC, respectively, whereas they were little affected by GSR and the type of significance thresholding. The event‐related GLM design appears to be more sensitive to task‐evoked modulations of EC, but provides model parameters with lower certainty than the block‐based design, while the latter is more sensitive to the type of activation contrast than is the event‐related design. Our results demonstrate that applying different reasonable data processing choices can substantially alter task‐evoked EC as estimated by DCM. Such choices should be made with care and, whenever possible, varied across parallel analyses to evaluate their impact and identify potential convergence for robust outcomes.

## Graphical abstract

This study considered the impact of data processing conditions on the task‐evoked modulatory effective connectivity (EC) using a stimulus–response compatibility task. Our study demonstrated that task‐evoked EC and parameter certainty were significantly impacted by the general linear model design and type of activation contrast as revealed by parametric empirical Bayes and Bayesian data comparison, respectively.

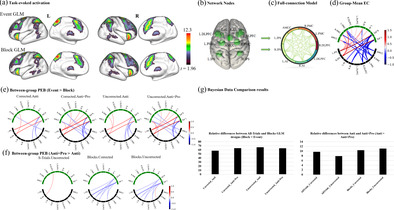

## 1 INTRODUCTION

One of the main approaches to studying the human brain consists in representing it as a collection of complex networks involving sets of brain areas engaged in different functions and continuously sharing information within and between the networks (van den Heuvel & Hulshoff Pol, 2010). In the framework of functional connectivity (FC), brain areas showing high temporal co‐activations are defined as functional networks during tasks or resting state (Menon, 2011). Both task‐evoked and resting‐state FC of functional magnetic resonance imaging (fMRI) have shown high similarities to each other as reported by several papers (Beheshtian et al., 2021; Cole et al., 2014; Cole et al., 2016; Heckner et al., 2021), see also a recent review (Bernstein‐Eliav & Tavor, 2024). Withal, the current FC studies frequently focused on the resting state (Greene et al., 2018), which has widely been used to investigate brain organization (Eickhoff et al., 2018; Yeo et al., 2011) and brain–behavior relationships (Biswal et al., 2010; Shen et al., 2017). However, the lack of external reference time points (e.g., stimulus onsets) and the absence of control over mental processes (Cole et al., 2016) as well as the typical FC calculation approach (Pearson correlation) limit the application of resting‐state FC to dynamic interactions evoked by contextual modulation.

Task‐evoked effective connectivity (EC) is supposed to estimate the directional or causal information flow among network nodes modulated by task demands (Friston et al., 2003). For this purpose, the dynamic causal modeling (DCM) approach was developed and is firmly established in neuroimaging research (Frässle et al., 2017; Frässle et al., 2018; Friston et al., 2014). Task‐evoked EC estimated by DCM was shown to reflect the interregional directional information flows (Friston, 2011; Friston et al., 2003; Menon, 2011; Menon & Uddin, 2010) and has been linked to human cognitive and executive performance in tasks such as finger tapping, working memory, response conflict resolution, reading, and so forth (Boudrias et al., 2012; Cieslik et al., 2011; Jung et al., 2018; Kahan et al., 2019; Loehrer et al., 2016; Morken et al., 2017; Volz et al., 2015).

Despite the success and relevance of DCM‐based estimates of EC, the impact of variations in data processing parameters on DCM outcomes has not consistently been addressed. For task‐evoked brain activity, it has been demonstrated that the present analytical flexibility in the field can have substantial effects on the reported results and, thus, on the reproducibility of neuroimaging findings (Botvinik‐Nezer et al., 2020; Carp, 2012). Similarly, the influence of data processing varieties has also been a topic of intense discussion in studies on FC (Cole et al., 2010; Power et al., 2014; Power et al., 2017; Smith et al., 2013). However, issues and challenges of analytical flexibility in estimating task‐evoked EC have rather been neglected so far and call for further investigation. Here we therefore focus on four important aspects of data processing in a typical DCM analysis.

The preprocessing of fMRI data concentrates on the cleaning of the acquired data from noise, which is essential for an appropriate extraction of the signals (Churchill et al., 2015). Typically, the cleaning includes several steps such as slice‐timing correction (Parker & Razlighi, 2019; Sladky et al., 2011), motion correction (Friston et al., 1996; Yan et al., 2013), nuisance regression (Liu et al., 2017; Power et al., 2017), temporal filtering (Davey et al., 2013), and spatial smoothing (Friston et al., 2000). Of these, global signal regression (GSR) has received much attention as a nuisance variable with a substantial impact on estimates of FC (Murphy & Fox, 2017) and will therefore be examined for its influence on task‐evoked EC in this study. In particular, GSR has been thought to remove physiological noise (Power et al., 2017) and help to detect significant FC (Fox et al., 2009; Varikuti et al., 2017). However, the application of GSR is controversial and may potentially distort activation and connectivity measures in the network‐specific ways (Anderson et al., 2011; Glasser et al., 2018; Murphy et al., 2009; Saad et al., 2012). Furthermore, the impact of GSR on resting‐state FC was often assumed to be major (Murphy & Fox, 2017), while GSR was recently shown to have only a minor influence on resting‐state EC estimations (Almgren et al., 2020). As its impact on task‐evoked EC has remained unclear, we investigated it in the present study.

Another important methodological issue pertains to the question of which design of the general linear model (GLM) is optimal for subsequently analyzing task‐evoked EC. According to presentations and types of task stimuli, block‐ and event‐related designs have been used to model brain blood‐oxygen‐level‐dependent (BOLD) signals to task events by convoluting the temporal function of their occurrence with the hemodynamic response function (HRF) (Buxton et al., 2004). The block‐based design aggregates multiple (similar) events into blocks to maximize hemodynamic responses of engaged brain regions during the same experimental conditions (Logothetis, 2008). The event‐related design models discrete events separately from each other and analyzes brain responses to individual events independently (Huettel, 2012). The choice of GLM design has not only been shown to impact task‐evoked activation and FC (Friston et al., 1999; Liu et al., 2001), but also the model selection in DCM (Daunizeau et al., 2011). However, the immediate impact of GLM design type (block‐ vs. event‐related) on task‐evoked EC has not been explored yet, which is why we addressed it in this study.

Besides the type of design, there are at least two more factors in the analysis of task‐fMRI data that may influence EC estimates derived from DCM: significance thresholding of voxels at the level of individual subjects and the choice of activation contrast of interest. The selection of significance thresholding methods at the group level impacted the data‐analytical stability of fMRI results (Botvinik‐Nezer et al., 2020; Roels et al., 2015). However, the significance thresholding at the individual level and its impact on task‐evoked EC have not appropriately been discussed yet. The activation contrast indicates the brain activation driven by a specific task condition and reflects the context‐dependent task‐evoked EC (Zeidman, Jafarian, Corbin, et al., 2019). Previous studies have already demonstrated that DCM estimated different task‐evoked modulatory EC (M‐EC) with selected network nodes if various contrasts were specified as modulatory inputs (Kuhnke et al., 2021; Ma et al., 2014). However, it is still unknown how M‐EC is statistically changed when different contrasts are considered for time series extraction and used to define the modulatory inputs in DCM analyses.

Based on these considerations, our study aimed to investigate the impact of GSR, GLM design, significance thresholding, and activation contrasts on task‐evoked EC. The main objective was to illustrate how important choices made during data processing can influence the results of the task‐evoked fMRI analysis and DCM estimations of the task‐based EC on an example of the stimulus–response compatibility (SRC) task (Fitts & Deininger, 1954). The workflow included several steps: (1) preprocessing task‐evoked images and reconstructing the SRC network nodes with different conditions of data processing (GSR and GLM designs); (2) extracting the respective BOLD time series from the SRC network nodes for individual subjects under different conditions with respect to GSR, GLM design, significance thresholding, and activation contrasts; (3) calculating the individual and group‐averaged task‐evoked EC patterns for each data processing condition; and (4) evaluating between‐group differences in task‐evoked EC as well as relative differences in EC parameter certainty between any two conditions of the data processing (with vs. without GSR, event‐related vs. block‐based designs, corrected vs. uncorrected thresholding, and whole task vs. incompatible contrasts). We show that different data processing choices result in substantially different task‐evoked EC at the group level, especially for the factors of GLM design and activation contrast. The obtained results could be of relevance for evaluating analytical flexibility in task‐evoked EC estimations.

## 2 METHODS

### 2.1 Participants and fMRI data

Our study included an initial sample of 271 subjects (148 males, 123 females, 18–85 years old, mean age: 52.3 ± 16.6 years) recruited from the subject pool of the 1000BRAINS project (Caspers et al., 2014), which was conducted at the Research Centre Jülich. Before MRI data collection, the written informed consent of each subject was acquired. The study protocol was approved by the health care ethics committee of the University Duisburg‐Essen (reference number: 11‐4678). The study was approved by the local ethics committee and performed in accordance with the declaration of Helsinki.

Details about fMRI data included in the 1000BRAINS project can be found elsewhere (Caspers et al., 2014). In the present study, only selected structural MRI (sMRI) and task‐based fMRI (t‐fMRI) data were used for analyses. Both sMRI and fMRI datasets were acquired on a 3‐T Siemens scanner (Tim‐TRIO, Siemens Medical System, Erlangen, Germany). The sMRI scans were obtained using an anatomical 3D T1w MPRAGE sequence with the following parameters: repetition time (TR) = 2.0 s, echo time (TE) = 3.03 ms, flip angle = 9°, 176 sagittal slices, field of view = 256 × 256 mm^2^, voxel resolution = 1 × 1 × 1 mm^3^. The t‐fMRI dataset was scanned by gradient‐echo echo‐planar imaging sequence with the following parameters: TR = 2.03 s, TE = 30 ms, flip angle = 80°, field of view = 200 mm, 33 axial slices (ascending), slice thickness = 3.3 mm, inter‐slice gap = 0.66 mm, voxel resolution = 3.1 × 3.1 × 3.3 mm^3^, acquisition time = 27 min, and 10 s.

### 2.2 Experimental protocol

**FIGURE 1:**
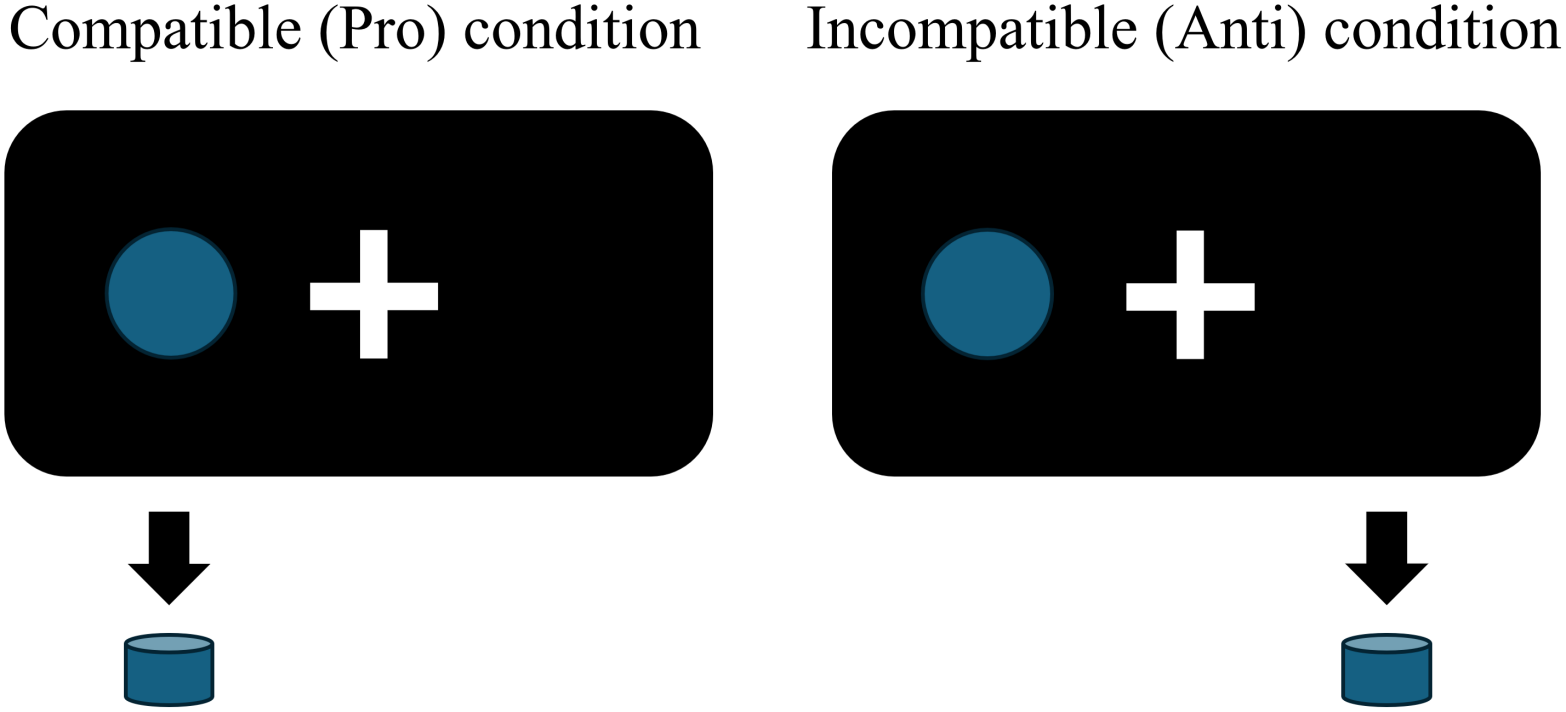
Schematic illustration of the spatial stimulus–response compatibility (SRC) task. A lateral stimulus on the screen (blue circle) called for a button press either on the ipsilateral or contralateral side, which is referred to as compatible (Pro) or incompatible (Anti) experimental condition, respectively.

The present study followed the standard spatial SRC paradigm (Fitts & Deininger, 1954). In particular, participants were required to respond to lateralized visual stimuli by pressing an ipsilateral or contralateral button as correctly and fast as possible (Figure 1). The whole experiment had 24 blocks and consisted of incompatible (Anti) and compatible (Pro) conditions. The Anti‐condition required participants to react to the lateralized stimulus by pressing the opposite button, while the Pro‐condition required participants to press the ipsilateral button. Before one block started, a 2‐s instruction was presented to indicate the condition (incompatible or compatible) of the following block. Each block contained 13 to 16 trials, in which filled circles (see Figure 1) were presented for 0.2 s either on the left or right side of the screen with an equal probability (50%) to be on either side. The time intervals between event onsets were uniformly jittered from 2 to 4.5 s. The rest periods between blocks were randomly jittered by a uniform distribution ranging from 15 to 19 s. Either experimental condition was covered in 12 blocks, which were presented in a pseudo‐randomized order with a stochastic paradigm.

### 2.3 Preprocessing

The sMRI and fMRI images were preprocessed using functions from FSL (Jenkinson et al., 2012), ANTs (Tustison et al., 2014), Workbench (Glasser et al., 2013), and AFNI (Cox, 1996) software packages.

The sMRI preprocessing included the following steps: (1) reorientation and cropping (functions *fslreorient2std* and *robustfov*) (Glasser et al., 2013), (2) AC‐PC alignment (*flirt*) (Glasser et al., 2013), (3) brain extraction (*antsBrainExtraction*) (Esteban et al., 2019; Tustison et al., 2010), (4) tissue segmentation of gray matter (GM), cerebrospinal fluid (CSF), and white matter (WM) (*fast*) (Zhang et al., 2001), and (6) nonlinear spatial normalization (*antsRegistration*) (Avants et al., 2008).

The fMRI module included the following steps: (1) removal of four dummy volumes (*fslroi*); (2) two‐pass head‐motion correction, which initially realigned all time points to the first volume, and subsequently to the averaged realigned volumes (*mcflirt*) (Jenkinson et al., 2002); (3) intensity normalization (scaled to 10,000; *fslmaths*); (4) co‐registration between the averaged functional volume and structural images (*antsRegistration*) (Avants et al., 2008); (5) functional normalization using the structural normalization matrix (*antsApplyTransForms*); (6) spatial smoothing with an 8‐mm full‐width at half‐maximum Gaussian kernel (*wb_command*) (Glasser et al., 2013); (7) regression of 27 nuisance regressors comprising 24 motion parameters (Friston et al., 1996) as well as the global signal of the whole brain, WM, and CSF (*fsl_glm*), and 8) high‐pass temporal filtering (cut‐off at 128 s, *fslmaths*) (https://fsl.fmrib.ox.ac.uk/fsl/fslwiki/). To study the impact of GSR, we applied an alternative pipeline without GSR in step 7 and regressed out only the other 26 regressors.

Our pipeline of data preprocessing mainly included specific functions of FSL, AFNI, and ANTs software, and selected functions were recommended by previous literature (Carp, 2012) as well as tested on the used dataset for high‐quality data processing. Our choice of structural and functional preprocessing modules was based on recommendations of HCP and fMRIprep pipelines (Esteban et al., 2019; Glasser et al., 2013), and applied a FEAT‐based statistical approach of FSL for extracting activation contrasts (Woolrich et al., 2004).

After a quality check of all preprocessing steps, 5 subjects were excluded because of bad quality in the spatial normalization, and 266 subjects were included in the subsequent analyses.

### 2.4 First‐level fMRI statistics

To model brain activation in response to task conditions as reflected by the dynamics of BOLD signals, we considered both event‐related and block‐based designs of the GLM (Woolrich et al., 2004) (FSL/*film_gls*). Our experimental protocol was designed in a way such that the BOLD signal could be modeled at the level of individual trials or blocks, and the experimental conditions of interest were modeled in the GLM in three different ways (1) event‐related model using all trials (All‐Trials), (2) event‐related model using only “successful” trials (i.e., trials with correct responses; S‐Trials), or (3) blocked design (Blocks). Thus, both All‐Trials and S‐Trials cases represent event‐related designs, but the S‐Trials design excluded the error trials, where subjects gave incorrect responses to stimuli (i.e., wrong response lateralization) or responded too fast or too slowly (reaction time, RT < 150 ms or RT > 1500 ms) or did not respond at all. The trials were considered in the framework of a given activation contrast of the investigated compatible/incompatible experimental conditions, see below. The explanatory variables of the event‐related GLM included the on–off step functions starting at the onset time of each trial with a fixed “on” duration of 0.2 s of the stimulus length (an example can be seen in Supplementary Figure S1). The block‐based GLM, in turn, used the starting time and full length of each of the 24 experimental blocks as onset and duration times, respectively, of the step function of the explanatory variables. The event‐related design had four regressors of interest comprising compatible and incompatible conditions with right‐ and left‐sided stimulus presentation, respectively, while the block‐based design had only two regressors representing compatible and incompatible blocks of trials.

After task designs had been specified, the double‐gamma HRF and their temporal derivatives were modeled to estimate whole‐brain voxel‐wise BOLD responses to the abovementioned task events (Woolrich et al., 2004). We also included temporal derivatives of the task regressors in the GLM design matrix to accommodate slight variations in the timing of the HRF across the brain and improve the fit of the data (Woolrich et al., 2004).

After model estimation, we computed four task contrasts: incompatible condition (Anti), compatible condition (Pro), incompatible versus compatible condition (Anti > Pro) (subtracted contrasts, Anti ‐ Pro), and incompatible + compatible condition (Anti + Pro) (sum contrasts) in all GLMs (an example can be seen in supplementary Figure S1). The Anti ‐ Pro contrast aims to detect brain regions that are more sensitive to the Anti‐condition than to the Pro‐condition, whereas the Anti + Pro contrast aims to detect brain regions responding to either experimental condition.

### 2.5 Second‐level fMRI statistics

**FIGURE 2:**
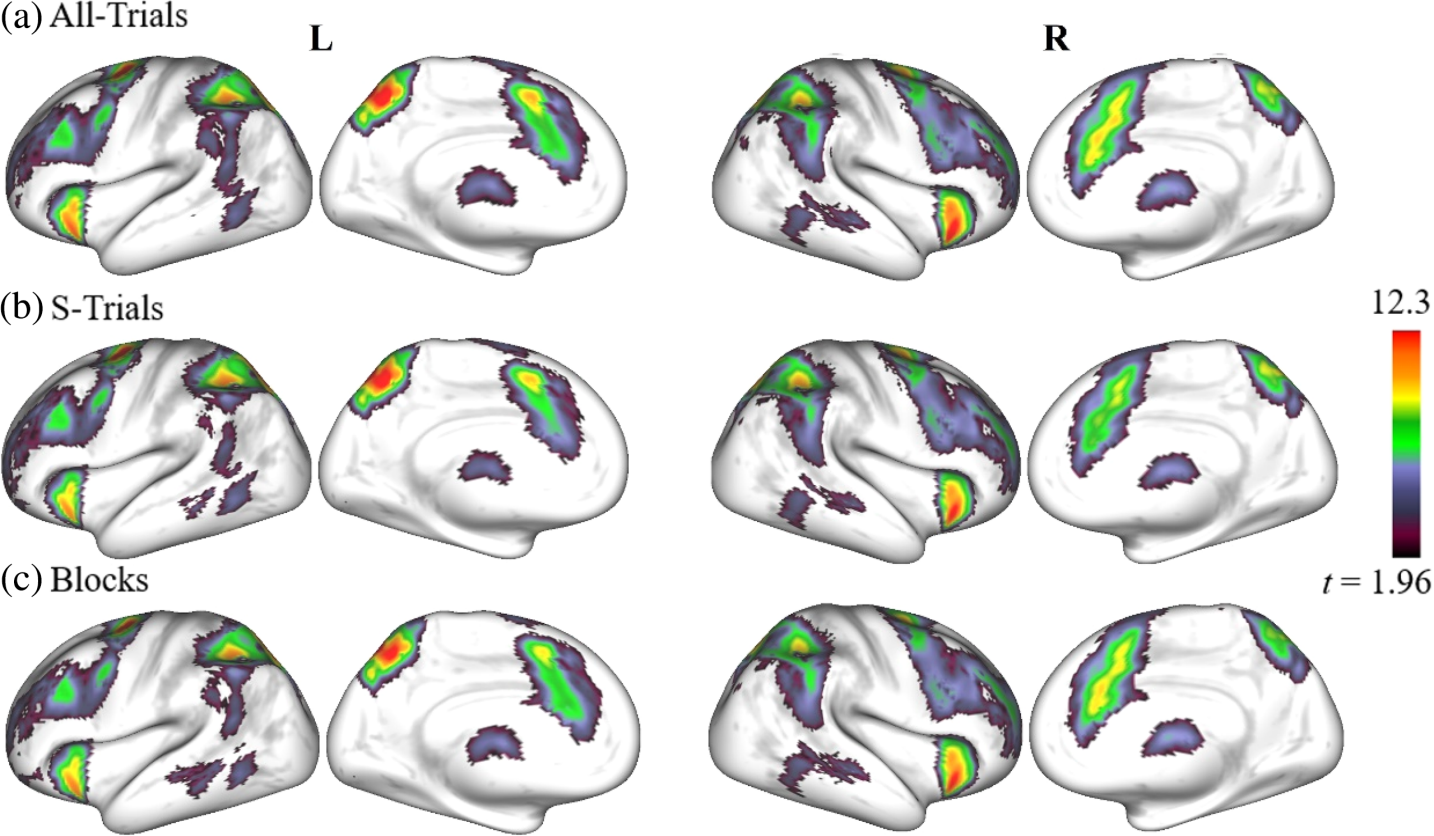
Results of the second‐level functional magnetic resonance imaging (fMRI) analysis with different general linear model (GLM) designs: (a) All‐Trials, (b) S‐Trials, and (c) Blocks designs (see text for details). All maps illustrate the *t*‐values (scaling is given in the color bar) of the *t* tests reflecting the statistically significant voxels across all subjects (*p*_TFCE+FWE_ < 0.05) of the contrast difference between incompatible and compatible experimental conditions (Anti > Pro contrast). For visualization, each thresholded statistical map was projected to fs_LR 32k surfaces (https://www.humanconnectome.org/software/connectome‐workbench). Used notations: L/R, left/right hemisphere; All‐/S‐Trials, experimental designs with all/successful trials; Blocks, experimental designs modeled by blocks; TFCE, threshold‐free cluster enhancement; FWE, family‐wise error.

To reconstruct the brain network activated during the SRC task at the group level, we calculated second‐level fMRI statistics for our different experimental designs using the FSL/*randomize* tool. The SRC paradigm aims to elucidate brain activity related to solving response conflicts arising from spatial incompatibility, which is why the Anti‐Pro contrast would be the most appropriate for network detection that was activated stronger at the spatial incompatibility condition, as compared to the other contrasts discussed. For reconstructing the incompatibility‐related brain network, the following steps were performed during the second‐level analysis of the fMRI data: (1) Contrast maps (Anti > Pro) of individual subjects were merged into 4D images for all subjects, and a one‐sample permutation test (Winkler et al., 2014) was conducted 10,000 times for All‐Trials, S‐Trials, and Blocks designs separately. (2) Threshold‐free cluster enhancement (TFCE) with family‐wise error (FWE) correction (Smith & Nichols, 2009) was applied for dealing with the issue of multiple comparisons (*p*_TFCE+FWE_ < 0.05). This resulted in several clusters of brain voxels demonstrating significantly stronger responses during the Anti‐condition than during the Pro‐condition (significant positive differences between Anti and Pro conditions) across subjects. The second‐level statistical maps are illustrated in Figure 2 for all three GLM designs with GSR; the cases without GSR are illustrated in Supplementary Figure S2.

### 2.6 Task‐evoked network and individual time series extraction

**FIGURE 3:**
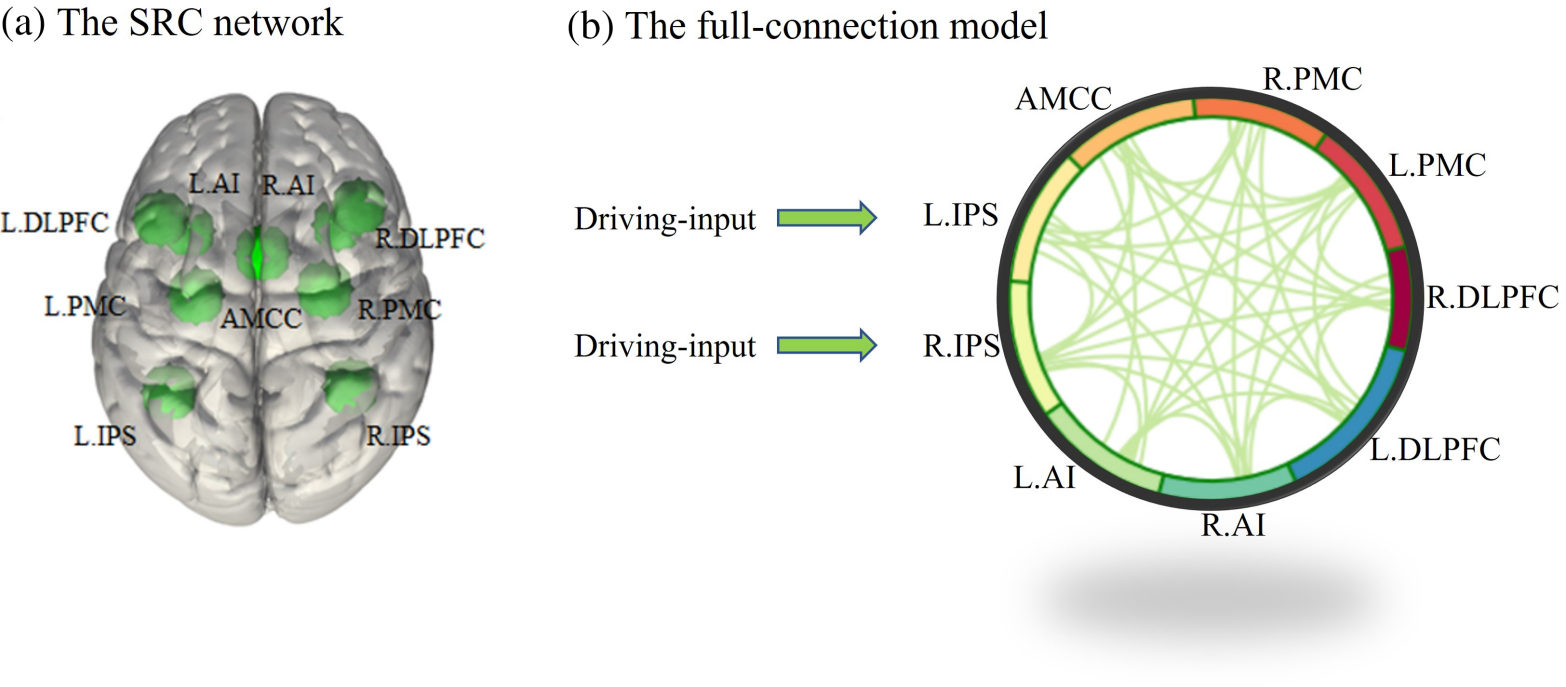
Illustration of the stimulus–response compatibility (SRC) network. (a) An example SRC network with nine nodes for the event‐related general linear model design, where the peak coordinates from Table 1 are encircled by spheres of a 10‐mm radius. (b) The corresponding full‐connection model used in dynamic causal modeling (DCM), see text for the node abbreviations. LIPS and RIPS are the driving‐input nodes receiving external (visual) stimuli of the task, while all connections inside the SRC network are bidirectional. Used notations: L/R, left/right; DLPFC, dorsolateral prefrontal cortex; PMC, premotor cortex; IPS, intraparietal sulcus; AI, anterior insula; AMCC, anterior midcingulate cortex.

After the second‐level fMRI statistics were completed, the local maxima of the group‐level Anti‐Pro contrast map were identified using the SPM 12 (v7219) package (http://www.fil.ion.ucl.ac.uk/spm/). Consistent with previous literature (Cieslik et al., 2010; Langner et al., 2015), we detected 9 regions of the task‐evoked brain network as major constituents: anterior midcingulate cortex (AMCC), bilateral intraparietal sulcus (IPS), premotor cortex (PMC), dorsolateral prefrontal cortex (DLPFC), and anterior insula (AI). These brain regions were selected to reconstruct the SRC network for the Anti‐Pro contrast. The Montreal Neurological Institute (MNI) peak coordinates of the second‐level statistical maps and the corresponding *t*‐values are given in Table 1 for all three GLM designs after GSR. Examples of the spheres (10‐mm radius) encircled around the corresponding peaks and representing the SRC network nodes (regions of interest [ROI]) are illustrated in Figure 3a.

**TABLE 1:** MNI peak coordinates (*x, y, z*) of the local maxima of *t*‐values based on the second‐level fMRI statistics of the Anti‐Pro contrast with global signal regression.

Peak	All‐Trials				S‐Trials				Blocks			
	*x*	*y*	*z*	*t*	*x*	*y*	*z*	*t*	*x*	*y*	*z*	*t*
LDLPFC	−40	22	28	**7.2**	−44	22	30	**7.0**	−40	22	28	**6.9**
RDLPFC	36	30	28	**6.7**	36	30	28	**6.7**	36	26	24	**5.9**
LPMC	−24	−8	48	**16.3**	−24	−8	48	**16.6**	−24	−8	48	**15.2**
RPMC	24	−8	48	**11.1**	24	−8	48	**11.4**	24	−6	48	**10.3**
LIPS	−34	−46	38	**11.1**	−34	−46	38	**11.0**	−34	−46	38	**10.9**
RIPS	36	−44	40	**10.7**	36	−44	40	**11.1**	36	−44	40	**10.2**
LAI	−30	18	−10	**12.1**	−30	18	−10	**11.0**	−32	18	−10	**11.6**
RAI	30	20	−4	**13.5**	30	20	−4	**13.0**	30	20	−6	**12.6**
AMCC	−2	8	46	**10.4**	−4	6	46	**10.0**	0	8	48	**9.7**

*Note*: Used notations: All‐/S‐Trials, experimental designs with all trials or only successful trials; Blocks, experimental designs modeled by blocks. Bold values are statistically significant.

Abbreviations: AI, anterior insula; AMCC, anterior midcingulate cortex; DLPFC, dorsolateral prefrontal cortex; IPS, intraparietal sulcus; L/R, left/right; PMC, premotor cortex.

MNI peak coordinates without GSR can be seen in Supplementary Table S1.

After SRC networks were reconstructed for the considered conditions of GLM designs and GSR, we focused on the extraction of BOLD signals of the network nodes reflecting the task‐evoked activity of individual subjects. The steps performed for time series extraction were the following:

1. The group‐level SRC network nodes (group‐level node ROIs: spheres of 10 mm radius centered at the peak coordinates from Table 1, see Figure 3a) were overlaid with thresholded contrast maps of individual subjects.
2. The local maxima and the corresponding voxel coordinates of the individual contrasts were searched for in the intersection between the group‐level node ROIs and individual thresholded maps.
3. For each network node, the detected coordinates of the individual local maxima were selected as centers of individual spheres with a 4‐mm radius, and these spheres were then considered as network nodes for individual subjects (subject‐level node ROIs).
4. Within every subject‐level node ROI, only voxels masked according to the individual thresholded contrast maps (see step 1) were considered, while the other voxels under the threshold were excluded. Then, the first eigenvariate was extracted from the BOLD signals of the masked significant voxels for every individual network ROI by using FSL/*fslmeants* and considered as time series of individual network nodes.

In step 1, we did not use the fixed network ROIs as observed at the group level but overlaid them with the thresholded contrast maps of individual subjects to ensure that the voxels used to summarize individual signals represented the task effects rather than irrelevant noise processes. If there was no overlap between individual thresholded contrast maps and the group‐level network ROIs, the respective subject was excluded from further analyses, as an empty intersection would lead to incomplete network reconstruction and BOLD extraction for individual subjects. As expected, the selected kind of significance thresholding of individual contrast maps strongly influenced the amount of overlap between individual activation clusters and group‐level SRC network nodes, with stricter thresholding reducing the sample size available for subsequent DCM analyses. Here, we applied two thresholding approaches to the considered contrasts for individual subjects with different levels of strictness: voxel‐wise thresholding with uncorrected *p* < .05 and cluster‐wise corrected *p* < .05 approaches, which we subsequently refer to as uncorrected and corrected thresholding, respectively. For the latter approach, cluster‐level inference was used to define contiguous voxels of individual thresholded maps by using FSL/*cluster*.

For the extraction of individual BOLD signals, four contrasts were considered in this study as candidates for voxel masking: Anti, Pro, Anti + Pro, and Anti ‐ Pro. Since the current study was focused on task‐evoked EC within the brain network showing incompatibility effects, we discarded the Pro contrast. Although we observed strong group‐based incompatibility effects as reflected by high *t*‐values of the second‐level statistics of the Anti‐Pro contrast (Table 1 and Figure 2), individual Anti ‐ Pro contrast maps did not display such a clear and pronounced activation (an example can be seen in Supplementary Figure S1). We found that individual Anti ‐ Pro contrasts yielded rather sparse and weak activation maps after significance thresholding such that many subjects did not qualify for further analyses as their individual thresholded Anti ‐ Pro contrast maps failed to overlap with the group‐level SRC network nodes. Aimed at the consideration of relatively large samples, we then discarded the Anti ‐ Pro contrast from further analysis. Hence, we applied the thresholding schemes mentioned above to individual Anti and Anti + Pro contrast maps for time series extraction for individual subjects (see Supplementary Figure S1). Therefore, four kinds of thresholded contrast maps were considered for individual time series extraction in this study: corrected Anti, corrected Anti + Pro, uncorrected Anti, and uncorrected Anti + Pro. Summary information on the participant samples that were included in subsequent analyses, after subject exclusions discussed above, can be found in Table 2 for the different contrasts and GLM designs with GSR (see Supplementary Table S2 for conditions without GSR).

**TABLE 2:** Sample sizes for different conditions of the data processing with GSR.

	All‐Trials		S‐Trials		Blocks	
	Corrected	Uncorrected	Corrected	Uncorrected	Corrected	Uncorrected
Anti	149/148	210/208	136/136	207/203	160/158	213/205
Anti + Pro	164/164	215/212	149/148	206/201	173/171	216/210

*Note*: The two subject numbers given in each table cell correspond to the subject samples qualified for BOLD signal extraction for SRC network nodes of individual subjects/explained variance (EV) criterion of DCM, see Sec. 2.6/Sec. 2.7 for details. Used notations: GSR, global signal regression; All‐/S‐Trials, experimental designs with all/successful trials; Blocks, experimental designs modeled by blocks; Anti, incompatible contrast; Anti + Pro, incompatible + compatible contrast.

The considered conditions of the data processing can be summarized as follows: (1) two GSR conditions, where the whole‐brain global signal was either regressed out or not (i.e., with or without GSR); (2) three GLM designs (i.e., All‐Trials, S‐Trials, and Blocks); (3) two individual first‐level brain activation contrasts of Anti and Anti + Pro used for BOLD signal extraction for SRC network nodes of individual subjects; and (4) two thresholding approaches for the individual contrasts based on either voxel‐level uncorrected *p*_*uncorr*_ < 0.05 or on cluster‐wise corrected *p*_*corr*_ < 0.05 thresholding. These conditions resulted in 2 × 3 × 2 × 2 = 24 cases of data processing investigated in this study.

### 2.7 Dynamic causal modeling

The present study evaluated task‐evoked EC within the SRC network via a two‐level DCM analysis (Zeidman, Jafarian, Seghier, et al., 2019) as implemented in SPM 12 (https://www.fil.ion.ucl.ac.uk/spm/). The DCM approach consists of approximating the neural mass dynamics *z*(*t*) by the following system of differential equations:

$$\frac{\mathrm{dz}}{\mathrm{dt}}=\left(A+\sum_{k}B^{\left(k\right)}u_{k}\left(t\right)\right)z+\mathrm{Cu}\left(t\right)$$

where the matrices *A* and $B^{\left(k\right)}$ stand for parameters of intrinsic and task‐modulated connectivity, respectively, and *u*_*k*_(*t*) encodes the timing of the experimental condition *k*. Matrix *C* represents the influence of all external experimental inputs (stimulation) *u*(*t*) on the neural dynamics of the considered ROIs.

At the first level, the DCM approach (Friston et al., 2003) was used to estimate the network‐based EC between the nodes of the SRC networks using the individual BOLD time series of the corresponding ROIs of individual subjects. The standard DCM analysis involves several parameters (Friston et al., 2003): (1) driving input that models external (e.g., visual) input to the network and forces the activity of the network nodes, and the input matrix C that defines the immediate influence of the driving input on the selected network nodes; (2) intrinsic connectivity (matrix A) that denotes task‐independent baseline connections among the nodes; and (3) modulatory connectivity (matrix B_j_) induced by the experimental (task‐dependent) condition decoded by variable *u*_*j*_ in the above equation and the respective cognitive processes. We also note that the u‐variables were not mean‐centered in the model, which allows us to interpret the A matrix as an intrinsic connectivity matrix, whereas all modulatory effects on EC due to experimental conditions are summarized in matrix B, as mentioned above (Zeidman, Jafarian, Seghier, et al., 2019).

One may observe that GLM designs (event‐related or block‐based) influence the activation contrast estimation and also the formulation of driving and modulatory inputs in the DCM model specification. For a consistent formulation of the driving and modulatory task‐dependent inputs to DCM, we followed the same formulation style throughout the GLM design, time series extraction for individual subjects, and DCM analysis (Supplementary Figure S3). For example, if the condition of the event‐related design and Anti contrast were considered for fMRI analysis and BOLD signal extraction, the driving and modulatory stimuli of DCM would also be event‐related, and the task‐evoked M‐EC would be driven by Anti trials only.

For investigating the impact of data processing parameters on task‐evoked EC within the SRC network, a full‐connection model was considered to be a good candidate (Tuominen et al., 2023). In the SRC network considered here, the IPS nodes were considered to act as hubs of sensorimotor integration during visually guided actions (Anderson et al., 2014), and the bilateral IPS nodes were thus selected as the driving‐input nodes receiving external (visual) input (Figure 3b). To compare the impact of the data processing conditions introduced above on the task‐evoked EC, we considered 24 DCM cases for every combination of data processing conditions mentioned above.

During the first‐level DCM analysis, where EC was estimated for individual subjects, we also evaluated the quality of the modeling and calculated the fraction of variance of empirical BOLD signals that can be explained by the variance of the simulated BOLD signals generated by the optimized models (i.e., for optimized connectivity matrices aimed at the best fit between empirical and simulated BOLD signals). In line with the literature (Zeidman, Jafarian, Corbin, et al., 2019), we applied a 10% threshold of the explained variance as a criterion for our subjects to qualify for DCM analysis. As a result, up to nine subjects had to be excluded from further analysis from those participants already qualified for BOLD extraction from the SRC network nodes of individual subjects, with the exact number depending on the selected data processing condition (see Table 2 and Supplementary Table S2).

For the second‐level DCM analysis, a parametric empirical Bayes (PEB) framework (Zeidman, Jafarian, Seghier, et al., 2019) was used to estimate the DCM parameters for group‐level EC. The PEB model can decompose the subject‐wise variability of EC into group effects and additive random effects (Friston et al., 2016). We adopted a two‐step PEB scheme involving single‐group and between‐group analyses (Zeidman, Jafarian, Seghier, et al., 2019). In the first step, we used the single‐group PEB analysis to investigate the group‐mean EC (commonalities) for each processing condition. In the second step, we applied the between‐group PEB analysis to analyze the differences of EC at the group level between the considered data processing conditions (i.e., the EC differences resulted from the application of any two different data processing conditions to the considered subject cohort). For both single‐ and between‐group PEB analyses, a 95% posterior probability (PP > 95%) threshold was taken as a strong evidence threshold rather than a statistical *p*‐value (Zeidman, Jafarian, Seghier, et al., 2019).

In parallel to PEB analyses, we also compared the relative difference in EC parameter certainty between any two processing conditions using Bayesian data comparison (BDC) as implemented in SPM12 v7771 (Zeidman, Kazan, Todd, et al., 2019). In contrast to Bayesian model selection, BDC allows for a systematic comparison between different datasets, such as those obtained from different data processing approaches as in this study. BDC analysis helps to make statistical inferences about the parameter certainty (reduction in uncertainty) of coupling parameters estimated for a given data set based on the relative entropy (Zeidman, Kazan, Todd, et al., 2019). A difference in the entropy between two data sets in the range between 1.1 and 3 nats (natural units of information) and between 3 and 5 nats can be considered as “positive evidence” and “strong evidence,” respectively, that the estimated parameters are more certain for one data set than for the other. A difference greater than 5 nats is indicative of “very strong evidence” (Tuominen et al., 2023; Zeidman, Kazan, Todd, et al., 2019). Based on this approach, we performed BDC analyses between two considered conditions with common subjects and extracted the relative differences in parameter certainty between them.

In our study, we focused on the impact of data processing conditions on the task‐evoked M‐EC (matrix B) within the SRC network. Based on the single‐group PEB analysis, we observed the group‐mean task‐evoked M‐EC for all conditions and identified varied EC patterns corresponding to different selections of data processing parameters. A systematic comparison was then performed directly between data processing conditions via between‐group PEB analysis.

## 3 RESULTS

In this study, we investigated the task‐evoked M‐EC (matrix B) depending on the condition of the data processing parameters (see Section 2). We considered 24 data processing conditions involving two GSR conditions, three GLM designs, two activation contrasts, and two significance thresholding methods. We investigated the impact of these conditions on the SRC network localization, analysis sample size, DCM model fits, task‐evoked M‐EC of matrix B, and its certainty as we illustrate below. Briefly, we observed that (1) variation of the data processing parameters resulted in varied group‐mean EC patterns; (2) the GLM designs and activation contrasts largely influence EC strength and parameter certainty; and (3) GSR and significance thresholding have a rather little impact on EC.

### 3.1 Task‐evoked network localization

Based on the second‐level fMRI analysis, the brain activation maps were obtained at the group level (Figure 2 and Supplementary Figure S2), and the peak coordinates of the SRC network nodes were determined (Table 1 and Supplementary Table S1). The data processing conditions of GLM design (All‐Trials, S‐Trials, and Blocks) and GSR (with/without) are relevant at this stage, and the remaining conditions of the activation contrast and significance thresholding will be applicable later at the time series extraction for the network nodes of individual subjects. When applying GSR, the results of the second‐level fMRI analysis were very similar across the three GLM designs with very high volumetric overlap as indicated by a large Dice coefficient D (Taha & Hanbury, 2015) (Supplementary Table S3). In particular, the overlap in the brain activation between the All‐Trials and Blocks cases was comparable with the overlap between All‐Trials and S‐Trials, with D = 0.94, respectively. We did, however, detect small differences in peak coordinates between GLM design types for the L.DLPFC, R.DLPFC, R.PMC, L.AI, R.AI, and AMCC nodes (between the All‐/S‐Trials and Blocks) and the L.DLPFC and AMCC nodes (between All‐Trials and S‐Trials). The factor GSR (i.e., with/without GSR) also showed a weak influence on the peak coordinates of the SRC network nodes: variations were observed in the R.PMC and R.AI nodes in Blocks, R.PMC, L.AI, and AMCC nodes in All‐Trials, and L.DLPFC, L.AI, and AMCC nodes in S‐Trials (compare Table 1 and Supplementary Table S1).

### 3.2 Analysis samples

Next, we examined the effects of processing conditions on the sample size of subjects available for subsequent DCM analysis. Different subject samples were qualified for individual time series extraction under different conditions of data processing. The type of significance thresholding (see Section 2) was found to be most relevant at this stage, as compared to the other three processing parameters considered. The sizes of the qualified subject samples are listed in Table 2 (left‐side numbers in the table cells), where the large impact of the significance thresholding can be seen. In many cases, the cluster‐corrected thresholding entailed excluding 50 more subjects than the uncorrected thresholding, which corresponded to more than 25% of the relative sample reduction. The choice of contrast (i.e., Anti vs. Anti + Pro) only slightly influenced the sample size in the range of 15 subjects. The factor of GLM design also weakly influenced the sample size of the qualified (or excluded) subjects, although the relative difference here reached up to 15% when comparing Blocks and S‐Trials designs (Table 2). The Blocks design entailed the largest sample qualified for time series extraction and subsequent DCM analyses, whereas the S‐Trials design led to the smallest sample eligible for further analyses. We replicated the above findings for the case without GSR (Supplementary Table S2).

### 3.3 DCM model fits

The goodness‐of‐fit of DCM can be evaluated by the fraction of variance of empirical BOLD signals that can be explained by the variance of the simulated BOLD signals generated by the model. Therefore, we calculated the fractions of the explained variance for all subjects qualified for BOLD signal extraction. We found that the DCM‐simulated BOLD signals can on average account for about 25% of the empirical variance (Supplementary Table S4). Only a few subjects (0–8) fell below 10% (Table 2). Varying the GSR condition (for other fixed conditions) also weakly affected the sample size with the differences in the range of nine subjects (Supplementary Table S2). Here, the differences between conditions were found to be statistically insignificant after multiple‐comparison corrections, and the modeling performed well for all conditions and most subjects.

### 3.4 Group‐mean EC estimation

**FIGURE 4:**
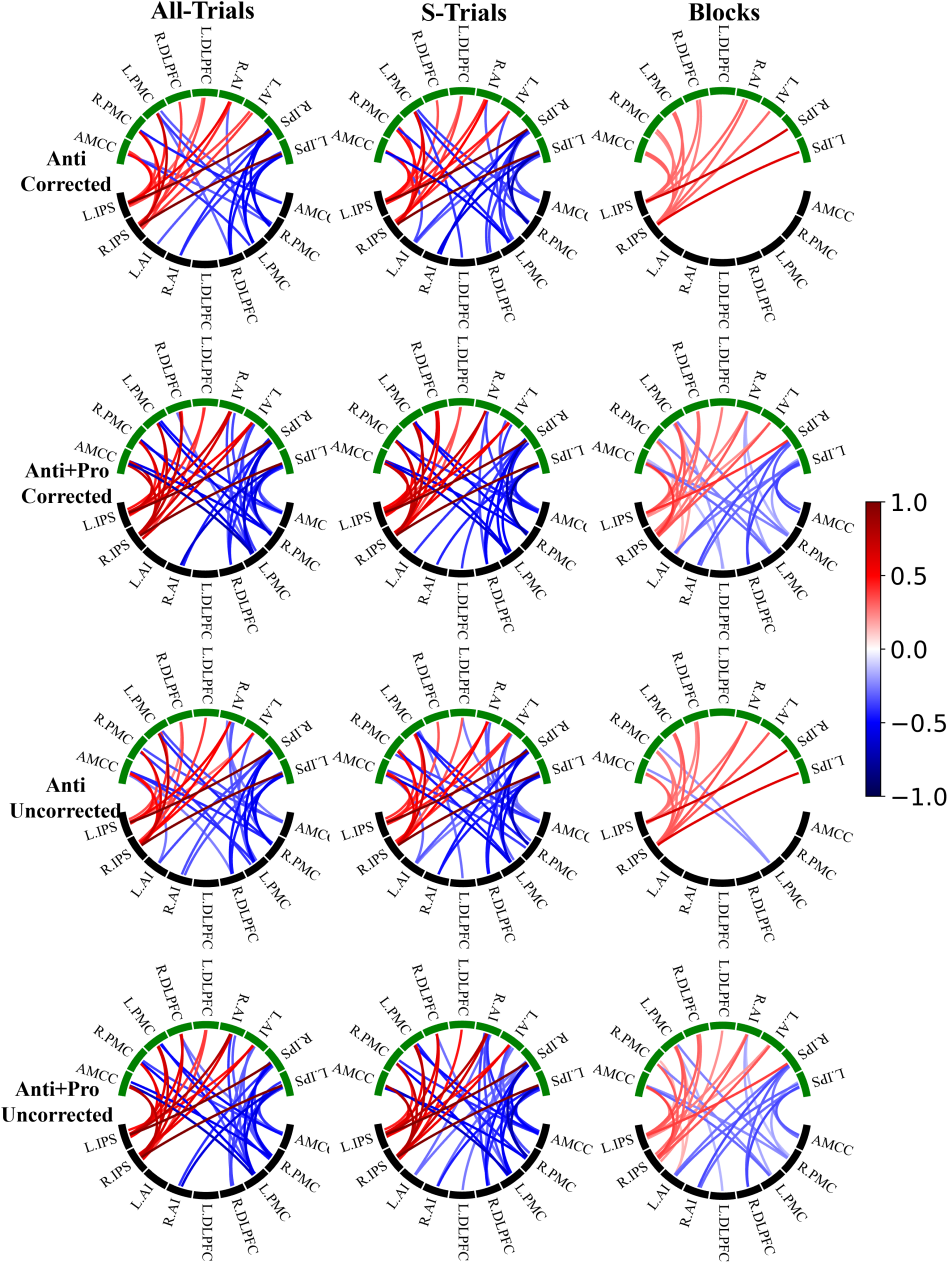
Group‐mean task‐evoked M‐EC (matrix B) for the considered conditions of the data processing indicated on the top and left sides of the circular plots with global signal regression (GSR) (see Section 2 for details and notations). The lower (black) and the upper (green) network nodes correspond to the sources (“from”) and destinations (“to”) of the illustrated directed connectivity, respectively. The values of the connectivity differences are reflected by color as indicated in the color bar. Only the M‐EC (PP > 95%) was displayed by connections in the circular maps. Used notations: All‐/S‐Trials, experimental designs with all/successful trials; Blocks, experimental designs modeled by blocks; Anti, incompatible contrast; Anti + Pro, incompatible + compatible contrast; L/R, left/right; DLPFC, dorsolateral prefrontal cortex; PMC, premotor cortex; IPS, intraparietal sulcus; AI, anterior insula; AMCC, anterior midcingulate cortex.

We estimated the averaged task‐evoked EC for considered data processing conditions (24 conditions) at the group level using the single‐group PEB analysis. We found that selecting one or another setup of the data processing influenced the results of DCM calculations and led to different group‐mean task‐evoked EC values. We first illustrate this by counting the numbers of evident edges (PP > 95%, see Section 2) of task‐evoked EC without counting self‐connections (Table 3). The edge number (PP > 95%) of the task‐evoked M‐EC (matrix B) was discovered to be varied depending on the selected approach of data processing. For example, the number of evident edges within the SRC network (in matrix B) may range from 42 (S‐Trials, uncorrected Anti + Pro) to 13 (Blocks, corrected Anti), which corresponds to a variation of the fraction of edges of the task‐evoked M‐EC of the SRC network from 58% to 18%, respectively (Table 3 and Figure 4).

**TABLE 3:** Numbers of the group‐level evident edges showing a high posterior probability of task‐evoked M‐EC (matrix B) within the SRC network.

	All‐Trials		S‐Trials		Blocks	
	Corrected	Uncorrected	Corrected	Uncorrected	Corrected	Uncorrected
Anti	31	36	35	39	13	15
Anti + Pro	38	39	37	42	33	32

*Note*: All task‐evoked EC exceeded the 95% posterior probability threshold (excluding self‐connections) and was calculated by the single‐group PEB analysis for the considered conditions of the data processing with GSR (see Section 2 for details and notations). Used notations: SRC, stimulus–response compatibility; All‐/S‐Trials, experimental designs with all/successful trials; Blocks, experimental designs modeled by blocks.

The choice of GLM design resulted in very different task‐evoked EC patterns, where the task‐evoked M‐EC of the Blocks‐design is much sparser than those of All‐Trials and S‐Trials designs (Figure 4 and Supplementary Figure S4). The uncorrected significance thresholding led to a denser task‐evoked EC compared with the corrected condition for the Anti activation contrast. The Anti + Pro contrast resulted in more evident edges of the task‐evoked EC than the Anti contrast did, except for the uncorrected All‐Trials‐condition. Without GSR application, small differences in task‐evoked EC were observed as compared to the case when GSR was applied (Figure 4, Table 3, Supplementary Figure S4, and Supplementary Table S5). Nevertheless, we corroborated the above conclusions also for the case without GSR.

### 3.5 Between‐group differences in task‐evoked EC

**FIGURE 5:**
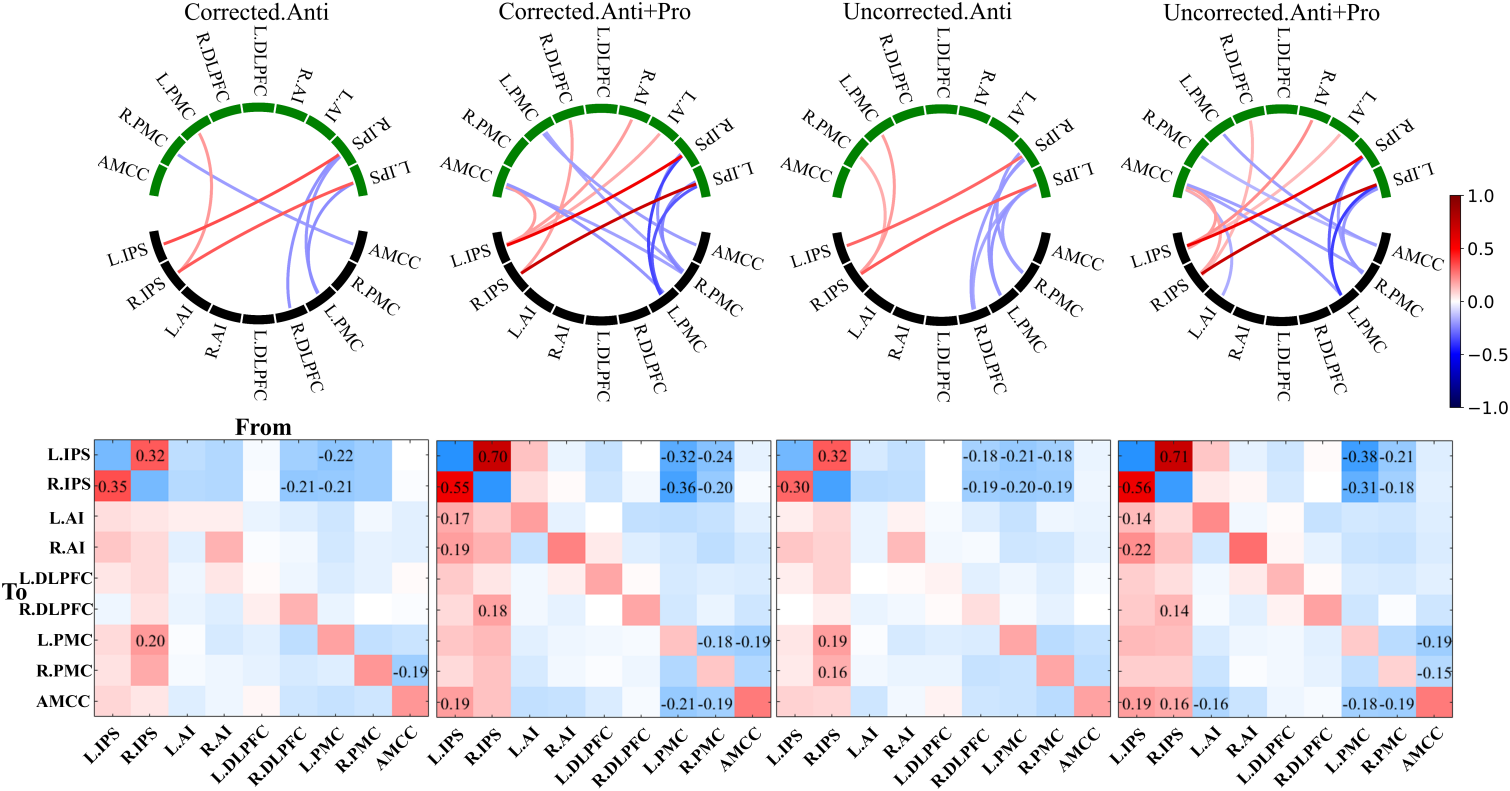
Comparison of task‐evoked modulation of effective connectivity (M‐EC) (matrix B) between the considered general linear model (GLM) designs (All‐Trials vs. Blocks). The results of PEB analyses at the group level are illustrated for the differences of B matrices, where the latter of Block designs was subtracted from that of All‐Trials designs (All‐Trials–Blocks). The other considered conditions of the data processing (contrast and thresholding) are indicated in the titles of the plots. In the circular network plots (upper row), the evident EC edges (PP > 95%) of the difference All‐Trials–Blocks are depicted. The lower (black) and the upper (green) network nodes correspond to the sources (“from”) and destinations (“to”) of the illustrated directed connectivity, respectively (see Section 2 for the nodes' abbreviations). The values of the M‐EC are reflected by color as indicated in the color bar. In the matrix plots (lower row), EC values are also depicted by color, and the values above PP > 95% threshold are indicated by numbers in the corresponding cells. The network nodes indicated in the horizontal and vertical axes correspond to the sources (“from”) and destinations (“to”) of the directed connectivity, respectively. Used notations: All‐/S‐Trials, experimental designs with all/successful trials; Blocks, experimental designs modeled by blocks; Anti, incompatible contrast; Anti + Pro, incompatible + compatible contrast; L/R, left/right; DLPFC, dorsolateral prefrontal cortex; PMC, premotor cortex; IPS, intraparietal sulcus; AI, anterior insula; AMCC, anterior midcingulate cortex.

**FIGURE 6:**
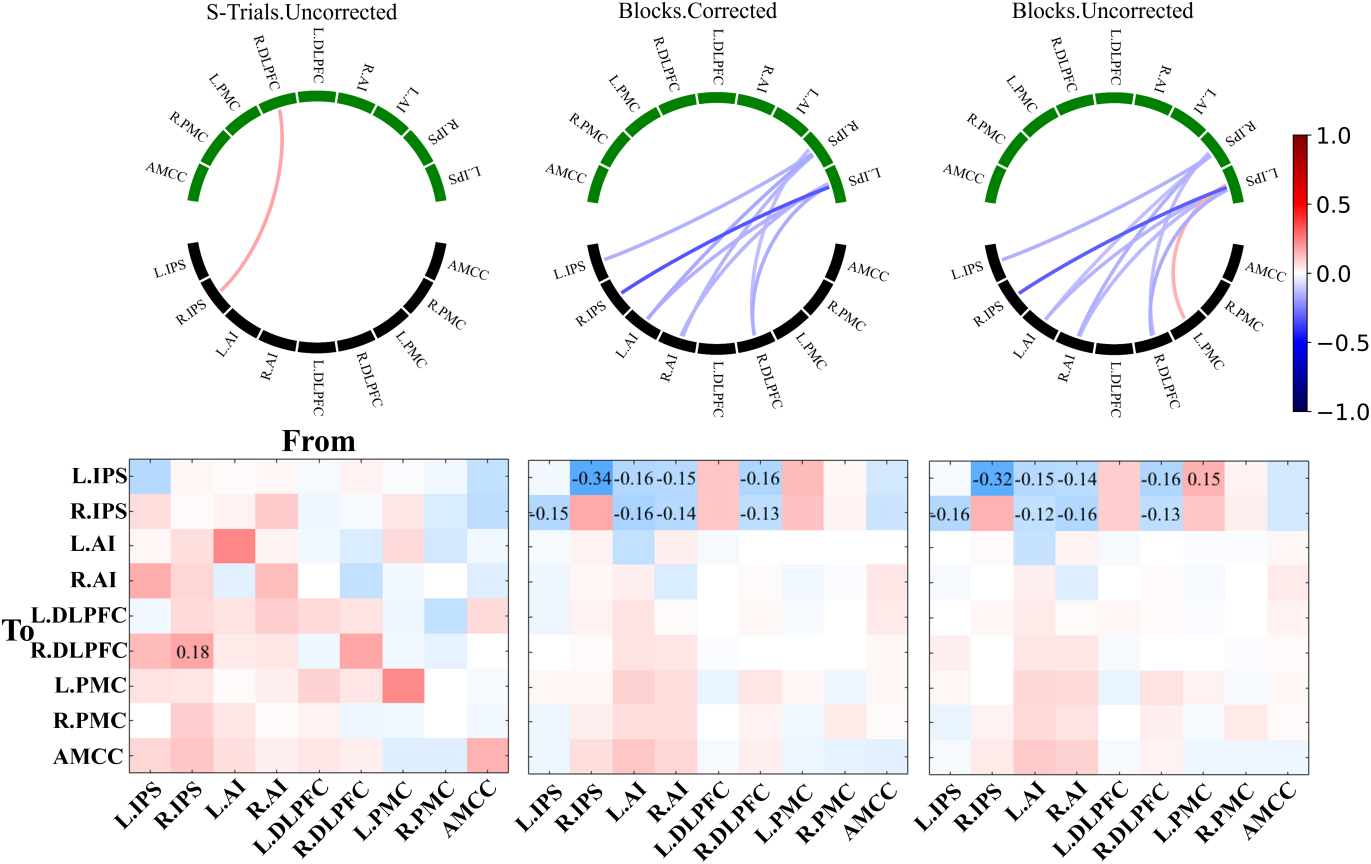
Comparison of task‐evoked M‐EC (matrix B) between the considered contrasts Anti + Pro and Anti. The results of parametric empirical Bayes (PEB) analyses at the group level are illustrated for the differences of B matrices, where the latter of the Anti contrast was subtracted from that of Anti + Pro‐contrast (Anti + Pro − Anti). The other considered conditions of the data processing (GLM design and thresholding) are indicated in the titles of the plots. In the circular network plots (upper row), the evident EC edges (PP > 95%) of the difference Anti + Pro − Anti are depicted. The lower (black) and the upper (green) network nodes correspond to the sources (“from”) and destinations (“to”) of the illustrated directed connectivity, respectively. The values of the modulatory connectivity are reflected by color as indicated in the color bar. In the matrix plots (lower row), EC values are also depicted by color, and the values above PP > 95% threshold are indicated by numbers in the corresponding cells. The network nodes indicated in the horizontal and vertical axes correspond to the sources (“from”) and destinations (“to”) of the directed connectivity, respectively. Used notations: All‐/S‐Trials, experimental designs with all/successful trials; Blocks, experimental designs modeled by blocks; Anti, incompatible contrast; Anti + Pro, incompatible + compatible contrast; L/R, left/right; DLPFC, dorsolateral prefrontal cortex; PMC, premotor cortex; IPS, intraparietal sulcus; AI, anterior insula; AMCC, anterior midcingulate cortex.

To evaluate the differences in the task‐evoked M‐EC (matrix B) between varied conditions of a given data processing parameter (i.e., All‐Trials vs. Blocks; with‐GSR vs. without‐GSR; corrected vs. uncorrected thresholding; Anti + Pro vs. Anti contrast), a between‐group PEB analysis (see Section 2) was applied. We found that the considered data processing conditions of the GLM design and activation contrast led to strongly different task‐evoked EC values (Figures 5 and 6, and Supplementary Figures S5 and S6), while EC was little affected by GSR application and thresholding approach (Supplementary Figures S7 and S8). Moreover, some M‐EC edges were discovered to be consistently present when combining group‐mean PEB and between‐group PEB analyses (Supplementary Figure S9). For example, four edges were observed to be stable between conditions of All‐Trials and Block GLM designs, while 10 EC edges were found to be stable between conditions of Anti + Pro and Anti contrasts.

### 3.6 Differences between block‐ and event‐related GLM designs

We observed strongly different patterns of the task‐evoked M‐EC (matrix B) between event‐related (All‐Trials and S‐Trials) and block‐based GLM (Figure 5 for All‐Trials vs. Blocks, Supplementary Figure S5 for S‐Trials vs. Blocks). All‐Trials design showed stronger positive modulation of the connections from the network nodes (L.IPS and R.IPS) receiving external (visual) driving inputs to the rest of the network. At the same time, these driving‐input nodes received stronger negative modulation of EC from the other network nodes for the All‐Trials design than for the Blocks design (Figure 5). The mentioned effects hold for both contrasts considered (Anti and Anti + Pro) and significance thresholding (corrected/uncorrected) conditions. However, the matrices of the differences (All‐Trials vs. Blocks) of the task‐evoked EC are sparser for the Anti contrast than for the Anti + Pro contrast, which indicates that more edges were strongly affected for the latter contrast by changing the GLM design between event‐related and block‐based ones. The Anti + Pro contrast may thus be considered as being more sensitive to the type of GLM design than is the Anti contrast (Figure 5). Analogously, by comparing the corrected and uncorrected thresholding used for individual BOLD extraction we found that the former (corrected) case appeared to be somewhat less sensitive to the selection of the GLM design (Figure 5).

Similar conclusions can be drawn from the comparison between S‐Trials and Blocks GLM designs, as illustrated in Supplementary Figure S5. Indeed, S‐Trails and Blocks designs resulted in strongly different task‐evoked M‐EC, where the Anti + Pro contrast is more sensitive to the variation of the GLM design than is the Anti contrast. Likewise, the uncorrected thresholding might be more sensitive to the GLM design for the Anti contrast, which is, however, not apparent for the Anti + Pro case (Supplementary Figure S5). Finally, we found no strong differences in group‐level task‐evoked EC between All‐Trials and S‐Trials GLM designs (Supplementary Figure S6). This is in contrast to the differences observed in the group‐mean EC (Figure 4), where the All‐Trials and S‐Trials GLM designs exhibited different connectivity within the SRC network. However, a detailed statistical analysis using the between‐group PEB analysis did not confirm the differential impact of these conditions on task‐evoked EC.

### 3.7 Impact of the task‐evoked activation contrasts

We observed strong effects of the considered contrasts (Anti and Anti + Pro) on task‐evoked EC in the between‐group PEB analysis (Figure 6). The main differences in EC for these contrasts were found in the edges coming from the rest of the network nodes to the driving‐input nodes (L.IPS and R.IPS). This phenomenon seems to be most pronounced for the Blocks design, whereas only one edge was affected for the S‐Trials design, which comes from the R.IPS node to the “internal” node R.DLPFC (Figure 6, leftmost column). The Anti + Pro and Anti contrasts led to different modulations between driving‐input nodes (L.IPS and R.IPS) and the rest of the network (Figure 6). The Blocks design appears to be more sensitive to the selection of one or another contrast, whereas the event‐related design was less affected by the contrast. The task‐evoked EC of the “internal” edges within the SRC network (i.e., excluding the input‐driven nodes L.IPS and R.IPS) appeared to be not affected by the contrast variability for all other data processing conditions considered.

### 3.8 Impact of GSR and significance thresholding

Different significance thresholding and GSR applications resulted in varied patterns of evident edges (PP > 95%) of group‐mean task‐evoked EC as indicated by the single‐group PEB analysis (Figure 4 and Supplementary Figure S4). However, there were no strong differences in the task‐evoked EC when the between‐group PEB analysis was performed for a more sophisticated comparison between the conditions of the significance thresholding and GSR (Supplementary Figures S7 and S8). We therefore conclude that the task‐evoked EC can be stable with respect to variations of the significance thresholding at the extraction of individual BOLD signals and the application of GSR.

### 3.9 Between‐group differences in parameter certainty

**FIGURE 7:**
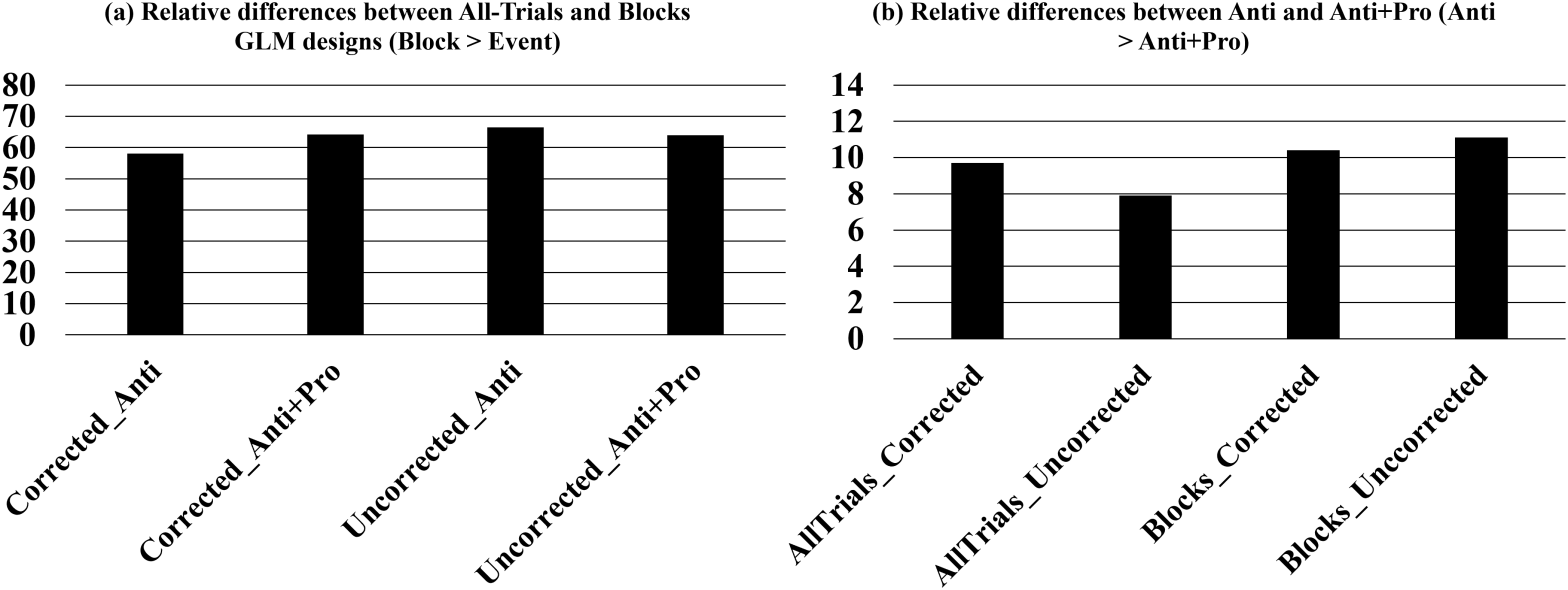
The parameter certainty of Bayesian data comparison (BDC) for two specific conditions. In panels (a) and (b), we present the BDC comparisons separately for (Block > All‐Trials) and (Anti > Anti + Pro), respectively. The Supplementary materials provide additional comparisons for conditions such as S‐Trials > Block and Anti > Anti + Pro of S‐Trials (Supplementary Table S6 and S7). The bar represents the relative differences in parameter certainty (negative entropy) between the conditions. Notably, the Block design exhibits a significantly higher parameter certainty compared to the All‐/S‐Trials design. Similarly, the Anti contrast demonstrates a notably stronger parameter certainty in comparison to the Anti + Pro contrast.

The between‐group BDC analyses demonstrated very strong evidence for differences in parameter certainty between conditions of GLM designs (Figure 7 and Supplementary Table S6) and between activation contrasts (Figure 7 and Supplementary Table S7). Block designs showed much higher parameter certainty than the event‐related designs (differences from 58 to 67 nats), but there was practically no evidence for a difference in parameter certainty between All‐Trials and S‐Trials cases (<1.1 nats except for the corrected Anti + Pro contrast with 2.3 nats). The Anti contrast displayed higher parameter certainty (from 7 to 11 nats) than the Anti + Pro contrast. No evidence was obtained for the certainty differences between GSR conditions (Supplementary Table S8) and between significance thresholding conditions (Supplementary Table S9), except for some evidence for the corrected Anti + Pro contrast in the All‐/S‐Trials case between GSR conditions (difference of 2.3 and 2.6 nats).

## 4 DISCUSSION

Our study examined the impact of several important data processing parameters on task‐evoked M‐EC within a brain network involved in solving spatial incompatibility‐induced response conflicts. In total, we considered 24 data processing conditions resulting from the combination of four factors: GSR, GLM design, activation contrast, and significance thresholding. In this study, we used the full‐connection model (i.e., with all connections between network nodes being equally admissible) to evaluate the EC estimates resulting from different data processing conditions, which ensured the same initial conditions for each DCM analysis (Tuominen et al., 2023). Furthermore, different data processing approaches investigated here can lead to altered time series even for the same subject, which might thus result in different optimal models for different cases. We therefore did not perform an exhaustive DCM model selection among potential SRC network topologies (by removal of specific connections) to infer a sparser model using Bayesian model reduction and selection approaches (Friston et al., 2016; Stephan et al., 2009). Instead, EC was calculated for the fully connected model of a network with nine nodes and then compared between different data processing conditions. Our study applied a two‐level DCM analysis that involved single‐ and between‐group PEB analyses as well as BDC. The single‐group PEB analysis showed that task‐evoked EC was sensitive to different choices of the considered data processing. The between‐group PEB analysis indicated that varying the type of GLM design and activation contrast may lead to strongly different task‐evoked EC and parameter certainty, whereas the connectivity and parameter certainty were little affected by GSR and significance thresholding.

### 4.1 Impact of GLM design

The fMRI analyses displayed consistent task‐evoked activation patterns (Figure 2 and Table 1) across considered GLM designs, while the two event‐related designs showed slightly higher peaks of *t‐*values. We note here that the employed event‐related GLM resolved the stimulus laterality of the experimental setup (Supplementary Figure S1), which cannot be accomplished for the block‐based design. We however confirmed that the event‐related GLM without modeling the laterality condition resulted in practically the same results in the second‐level analysis.

The single‐group PEB analysis showed that event‐related designs had a denser task‐evoked M‐EC than the block‐based design (Figure 4 and Table 3), which is consistently manifested in the interactive connections between driving‐input nodes and the “internal” nodes of the network. These connections may indicate that the experimental visual inputs could effectively exert influences on “internal” nodes (Friston et al., 2003). Although the event‐related GLM designs showed more strongly evident (with PP > 95%) modulatory connections than the block‐based design, the minimal number of evident EC edges in the latter case can still reach 18% (compared to 58% for the event‐related case) of the network capacity with 13 connections in the task‐evoked modulatory component of EC (matrix B) from 72 possible edges without self‐connections (Table 3). This may suggest that both types of GLM design can evoke M‐EC within the SRC network driven by task stimuli, although EC is more responsive to the task‐induced modulations for the even‐related GLM.

The between‐group PEB analysis further showed that strongly altered task‐evoked EC was associated with variations of GLM design. Here, the event‐related designs in most cases showed stronger positive and negative connections than did the block‐based design at the group level and thus stronger responses to the experiment (Figure 5). The strongly different edges mostly were the interactive connections between the driving‐input and “internal” nodes. This result agrees with other findings from the literature that experimental manipulations can perturb the brain's neural activities and EC parameters (Friston et al., 2003; Kahan & Foltynie, 2013). In contrast to our study, previous research (Daunizeau et al., 2011) attempted to find an optimized GLM design for a better model selection in DCM using the Laplace‐Chernoff risk, that is, a measure of model selection error rates. In this case, within‐subject experimental sessions were conducted with a block (consecutive identical trials) and an event‐related (randomized trials) design, and block‐based and event‐related Laplace‐Chernoff risks were compared. Although the experimental sessions included different trial‐presenting paradigms, and task‐evoked EC values were not compared with each other, the reported findings suggest that the type of GLM design can impact the DCM analyses, at least for the model selection (Daunizeau et al., 2011). The GLM design was also found to impact functional localizations in task‐evoked activation studies (Bühler et al., 2008; Tie et al., 2009), when the two GLM design types were compared directly. In particular, the event‐related design was found to lead to stronger activation and functional localization in putative language areas (Tie et al., 2009), while the block‐based design exhibited more activation in nonspecific areas (Bühler et al., 2008). The difference may have been caused by different shapes of the hemodynamic responses, when different GLM models were convolved with the HRF (Mechelli, Henson, et al., 2003; Mechelli, Price, et al., 2003). Here, the variance of the BOLD signal was better explained by GLM models of event‐related design, where the predicted hemodynamic responses reached the peak earlier but returned to baseline later (Mechelli, Henson, et al., 2003).

Our study analyzed data collected during an SRC task using a mixed block/event experimental protocol (Fitts & Deininger, 1954; Petersen & Dubis, 2012), which presents stimuli in a stochastic manner within blocks of trials with the same task set (i.e., respond with ipsilateral or contralateral button presses, respectively). On the one hand, this protocol is fair to compare both block‐based and event‐related GLMs. On the other hand, it reduced the anticipation effects and was able to extract transient activities in event‐related designs (Dosenbach et al., 2006). As mentioned above, the event‐related design went along with stronger positive and negative connections from the driving‐input nodes to “internal” nodes and backward, respectively, than what the block‐based design did. When examining the averaged absolute intensity of task‐evoked M‐EC (PP > 95%) for each condition (Supplementary Table S10), both All‐Trials and S‐Trials designs showed higher connectivity intensity than Blocks designs. The driving‐input connections may reflect the change rate of neural responses induced by the task stimuli presented (Kahan & Foltynie, 2013; Zeidman, Jafarian, Corbin, et al., 2019; Zeidman, Jafarian, Seghier, et al., 2019). The stronger positive and negative connections involving driving‐input nodes may suggest a higher responsivity of EC to task modulations in the event‐related designs than in the block‐based design. The EC sensitivity to the task‐induced modulations can be enhanced/reduced by other parameters of the data processing (see Table 3 and Figure 4). For example, EC for the block‐based design appeared to be more responsive to the task‐evoked modulations for the Anti + Pro contrast than for the Anti contrast.

While small numerical differences in M‐EC values were observed at the group level between All‐Trials and S‐Trials designs (Table 3 and Figure 4), no strong difference was detected by the two‐group PEB comparisons (Supplementary Figure S6). Comparing these GLM designs aimed at revealing a possible impact of including error trials in the analyses of task‐evoked fMRI data and EC. Usually, incorrect trials are regressed out or excluded from consideration before analysis (Ma et al., 2014; Zeidman, Jafarian, Corbin, et al., 2019), because incorrect trials are supposed to bring additional noise to the task‐driven data and may thus negatively affect the results. The small difference in EC between the All‐Trials and S‐Trials designs observed in our study might be due to the low rate of error trials of about 3% (Supplementary Figure S10) and the strict threshold for EC parameters (PP > 95%).

Consistent with our findings from connectivity strength comparisons, the BDC analyses also suggested very strong differences (nats >5) between event‐related and block‐based designs, but no difference between the different cases of event‐related designs (All‐Trials vs. S‐Trials). The parameter certainty of BDC reflects the confidence that we can place into estimated connections from a given model and is thought to be positively correlated to the signal‐to‐noise ratio (Zeidman, Kazan, Todd, et al., 2019). The stronger parameter certainty in the block‐based design may thus indicate a greater stability of EC estimates than obtained with event‐related designs. We also verified that the event‐related design resulted in broader posterior distributions of the M‐EC parameters, which, together with lower certainty, may indicate an enhanced variability of the parameters and noise in the event‐related modeling approach. Nevertheless, it is interesting to observe that the event‐related GLM and DCM designs led to a larger number of strongly evident (with PP > 95%) M‐EC parameters and stronger connectivity intensity than did the block‐based design (Figure 4). In our DCM model specification, block‐based designs included a longer time period of a constant experimental condition, which may reduce the effect of data variability and noise and may thus contribute to higher confidence about parameter estimates. On the other hand, fewer evident connections and smaller total modulated connectivity for the block‐based GLM design may also indicate a reduced sensitivity of this condition to the task‐evoked modulation of neuronal dynamics and connectivity as compared to the event‐related designs.

Furthermore, different GLM designs may better reflect different cognitive substrates, where the event‐related and block‐based designs can be more sensitive to transient and sustained brain activity, respectively (Petersen & Dubis, 2012; Visscher et al., 2003). The mentioned differences are, however, hardly reflected in the results of the neuroimaging analyses performed before DCM. For example, the brain activation maps strongly overlap for different processing conditions as reflected by large Dice coefficients and small differences in a few peak activation coordinates and their *t*‐values (Table 1, and Supplementary Tables S1 and S3). The task‐evoked brain activity extracted for individual subjects and used in DCM also exhibited a high similarity across different processing conditions. For example, the correlation between the BOLD signal time series of the event‐related and block‐based designs is larger than 0.9 (Supplementary Figure S11). The DCM was fitted equally well to these BOLD signals such that we cannot report any significant difference in the variance of the empirical data explained by DCM (Supplementary Table S4). Nevertheless, we found noticeably different task‐evoked EC estimates for different data processing conditions, in particular, between even‐related and block‐based GLM and DCM designs, which makes the reported results intriguing. This may indicate an enhanced sensitivity of the DCM approach, which seems to have picked up rather subtle differences in the empirical brain activation data and DCM design (event‐related and block‐based) and translated them to substantial EC differences.

The sensitivity of the DCM approach to the choice of design (event‐related vs. block‐based) was confirmed by a brief examination of the M‐EC obtained for the same BOLD time series extracted for individual subjects in the block‐based GLM case for the uncorrected Anti + Pro contrast (Supplementary Figure S12). We again observed very different connectivity patterns for the event‐related and block‐based DCM designs, which resemble the connectivity patterns illustrated in Supplementary Figure S4 for the group‐mean M‐EC and their differences in Figure 5. The same applies to the differences in parameter certainty as calculated by BDC (compare Figure 7a to Supplementary Figure S12d), although the input data in the latter case was the same, where we used the same BOLD time series but different DCM designs.

Jointly considering our findings regarding connectivity strength and parameter certainty, it is rather difficult to firmly conclude what type of design may (generally) be better for DCM analysis if both designs are equally reasonable to choose depending on the posed neuroscientific questions of the study. However, we systematically illustrated how different EC results can be for different GLM and DCM designs, highlighting the need for a sound rationale behind this impactful choice for any DCM analysis.

### 4.2 Impact of activation contrasts

The PEB analyses showed strong differences in task‐evoked EC between the Anti and Anti + Pro contrasts. The BDC analyses also revealed stronger parameter certainty (from 7.0 to 11.1 nats) for the Anti contrast relative to the Anti + Pro contrast (Supplementary Table S7). The Anti contrast reflects brain activation in response to incompatible trials, while the Anti + Pro contrast reflects brain responses to both incompatible and compatible trials. Psychologically, both Anti and Anti + Pro contrasts reflect a range of SRC task‐related processes that comprise stimulus identification, attentional orientation, response selection including inhibition of the inadequate response tendency elicited in incompatible trials, and response execution (Cieslik et al., 2010). In contrast to the Anti + Pro sum contrast, the Anti contrast is more specifically focused on incompatibility‐related processes (Munoz & Everling, 2004; Nee et al., 2007; Reuter‐Lorenz & Park, 2010). M‐EC is context‐dependent, and the selection of contrast in the DCM model can reflect the dynamics corresponding to specific cognitive or executive processes (Kuhnke et al., 2021). In our case, Anti + Pro and Anti contrasts showed different M‐EC patterns in group‐mean EC (Figure 4 and supplementary Figure S4). We further found that the Anti + Pro condition featured a stronger (positive and negative) modulatory connectivity between the driving‐input and “internal” nodes than did the Anti condition (Figure 6). Here, the sensitivity of EC to the contrast selection was additionally influenced by the type of GLM design, where the block‐based condition appeared to be more sensitive to the difference between Anti and Anti + Pro contrasts. The Anti + Pro contrast, in turn, featured an enhanced sensitivity of task‐evoked EC to the type of GLM design, especially when comparing event‐related and block‐based designs (see Figures 4 and 5). The difference in parameter certainty might be related to differences in signal variability between the two contrasts. As discussed above, the Anti + Pro contrast was assumed to reflect the averaged level of cognitive demands across all experimental conditions including compatible and incompatible cases (Figure 1), whereas considering the Anti contrast only was supposed to reflect states of higher cognitive demand arising from the need to solve incompatibility‐induced response conflicts. We may suspect that the inclusion of the Pro contrast may lead to overall stronger data variability and, thereby, lower certainty of the connectivity parameters.

### 4.3 GSR effects

We observed only small effects on the task‐evoked brain activation and group‐level EC induced by the application of GSR (Supplementary Figures S2 and S4). Accordingly, between‐group PEB comparisons found no strong differences in EC between the cases with/without GSR (Supplementary Figure S8). The global signal is supposed to capture physiological and motion‐related noise (Liu et al., 2017; Power et al., 2017) and the removal of the global signal is known to strongly influence resting‐state FC (Anderson et al., 2011; Fox et al., 2009; Murphy et al., 2009; Varikuti et al., 2017). However, regarding EC, a recent study (Almgren et al., 2020) reported only minor differences in within‐network EC estimates during the resting state before and after GSR. Our findings also agree with earlier studies in which the resting‐state FC retained its significant coupling (Chang et al., 2009; Weissenbacher et al., 2009), and task‐evoked FC between functionally related areas was not substantially affected (Mascali et al., 2021) when GSR was applied. We also consistently observed a minor difference in parameter certainty (from −2.6 to 1.6 nats) between the conditions with and without GSR (Supplementary Table S8). However, the effect of the global signal may be influenced by other factors such as intensity normalization and spatial normalization methods. For instance, some studies found that signal intensity normalization and GSR may share a similar effect on fMRI data (Liu et al., 2017; Smith, 2004). Although they are two distinct preprocessing steps, the intensity normalization scales the signal to a common value that may potentially remove global signals as GSR is assumed to do. In the present study, we scaled images to a common value of 10,000, which may influence the effect of GSR. It might be interesting to see if other data processing steps, for example, linear and nonlinear spatial normalization can influence the impact of GSR on EC. Nevertheless, we observed a similarly weak impact of GSR in line with the results of (Almgren et al., 2020; Mascali et al., 2021), who used different data processing pipelines. However, the effect of the global signal in task‐evoked fMRI still needs more specific and deeper investigation.

### 4.4 Thresholding effects

The significance thresholding of the activation contrast maps influenced both the extraction of the individual regional BOLD signals and subject qualification for such a signal extraction. It was thus suspected to be an important parameter also for the estimation of task‐evoked EC. Indeed, the choice of corrected versus uncorrected thresholding strongly influenced the size of the sample available for subsequent DCM analyses (see Table 2). Moreover, the density of the modulatory components (matrix B) of task‐evoked EC was altered depending on the thresholding, especially for the Anti contrast, where the uncorrected thresholding led to more evident EC edges (Table 3 and Figure 4). Although sufficient sample sizes are important for the robustness and statistical power of neuroimaging analyses (Button et al., 2013), the proper sample size is not commonly determined (Guo et al., 2014). At some point, it has been suggested that for reaching sufficient statistical power, a sample size of 24 subjects would be required for fMRI activation studies (Desmond & Glover, 2002), while a sample size of at least 20 subjects was suggested for DCM studies (Thirion et al., 2007). However, these numbers depend on the effect size of interest, which in turn may be influenced by many factors including tasks, acquisition parameters, and participants (Goulden et al., 2012). From the side of reproducibility, the typical sample size (*n* = 100) may reach a modest degree of replicability for task fMRI studies (Turner et al., 2018), although the sample size for high reproducibility varied across different tasks (Bossier et al., 2020).

The impact of sample size on EC estimation was not investigated in our study, and we included samples of ~150 to 220 subjects, mainly depending on the thresholding approach. The variation of ~25% of subjects was found to have little impact on task‐evoked EC according to the between‐group PEB analysis. The first reason for this insensitivity may be the high probability threshold (PP > 95%) of our PEB analysis. We observed numerically different densities of group‐mean EC in conditions of corrected versus uncorrected contrasts, but the difference was not large enough for direct comparisons via PEB analysis to become strong (see Supplementary Figure S7). A second reason may be related to the thresholding itself. During the SRC network reconstructions for individual subjects, the cluster‐corrected (vs. uncorrected) thresholding resulted in fewer significant voxels, leading to empty network nodes when overlapping individual maps with the ROIs obtained from the second‐level analysis and, thereby, to subject disqualification for BOLD extraction. Different thresholding may not affect the voxels in the vicinity of peak coordinates, and the BOLD signals extracted for the subject samples qualified for both corrected and uncorrected thresholding hardly differed from each other (Supplementary Figure S4). This may be another reason why the sophisticated between‐group PEB analysis did not find any strong differences in M‐EC between these two conditions (Supplementary Figure S7).

The results from BDC also showed a very minor difference ranging from −0.2 to 1.1 nats in parameter certainty between corrected and uncorrected thresholding approaches (Supplementary Table S9). This was observed despite different sample sizes resulting from the two thresholding approaches. Our findings thus indicate that the choice of significance thresholding influenced the sample size but did not much impact task‐evoked EC.

### 4.5 Limitations

Some limitations should be considered. First of all, the trade‐off of using multiple software applications, such as FSL, ANTs, AFNI, and Workbench, instead of using a single software solution, such as SPM, might be considered. While this approach can increase functionality and flexibility and make it easier to be conducted in computational clusters, it may also increase complexity and potentially impact comparisons to a literature that used SPM throughout. To address this question, we used the spatially preprocessed images as described in the Methods and then applied the SPM‐based pipeline for the entire analysis of the task‐evoked fMRI data and EC calculation by DCM (see also Arias et al., 2021; Hofmann & Straube, 2019; Park et al., 2018), which combined non‐SPM pipelines for data processing, but estimated EC using the SPM functions. The application of the SPM pipeline largely confirmed our main conclusions with respect to the differences between event‐related and block‐based GLM and DCM designs with some quantitative distinctions (see Supplementary Figure S13, Supplementary Tables S11 and S12, and the pertinent discussion in the supplementary material).

The second possible limitation can be that the present study was initiated from the end‐user perspective and focused on EC changes when different data processing decisions were made. This may impact interpretations of results because our study was not designed to ask a statistically well‐formed question or a specific hypothesis testing, but focused on exploratory investigations. Third, the generality of our findings may be limited to the specific task paradigm and sample characteristics considered here, which may be evaluated in further studies.

## 5 CONCLUSION

This study investigated the impact of four important data processing choices on the results of task‐evoked fMRI analyses and EC estimations via DCM in the framework of the SRC task. Our results showed that the type of GLM design (event‐related or block‐based) and type of activation contrast strongly affect task‐dependent EC estimation. In contrast, the other two processing factors examined here, GSR application and significance thresholding, appear to have only a weak influence on within‐network task‐evoked EC estimation. The event‐related design may confer a higher responsivity of EC to task stimuli, while the block‐based design featured a higher sensitivity of EC to the type of activation contrast. Our findings showcase the differential impact that various data processing choices may have on the estimation of task‐evoked EC, highlighting the importance of thoroughly considering and further assessing these choices to help build better models that allow for valid neuroscientific interpretations.

## FUNDING INFORMATION

This work was supported by the Portfolio Theme Supercomputing and Modeling for the Human Brain by the Helmholtz Association, and the European Union's Horizon 2020 Research and Innovation Programme under Grant Agreements 945539 (HBP SGA3, DOI: 10.3030/945539) and 826421 (VirtualBrainCloud, DOI: 10.3030/826421). Open access publication was funded by the Deutsche Forschungsgemeinschaft (DFG, German Research Foundation)—491111487. The funders had no role in the study design, data collection, analysis, decision to publish, or preparation of the manuscript.

## CONFLICT OF INTEREST STATEMENT

The authors declare no conflict of interest.

## Supporting information


**DATA S1:** Supporting information.

## Data Availability

The raw data of the 1000BRAINS project used in this study are not immediately available for public sharing because the authors do not have permission to share data. The codes are available in the (https://github.com/CogPsycho2023/DCMs1).

## References

[hbm26751-bib-0001] Almgren, H. , Van de Steen, F. , Razi, A. , Friston, K. , & Marinazzo, D. (2020). The effect of global signal regression on DCM estimates of noise and effective connectivity from resting state fMRI. NeuroImage, 208, 116435. 10.1016/j.neuroimage.2019.116435 31816423 PMC7014820

[hbm26751-bib-0002] Anderson, B. A. , Laurent, P. A. , & Yantis, S. (2014). Value‐driven attentional priority signals in human basal ganglia and visual cortex. Brain Research, 1587, 88–96. 10.1016/j.brainres.2014.08.062 25171805 PMC4253668

[hbm26751-bib-0003] Anderson, J. S. , Druzgal, T. J. , Lopez‐Larson, M. , Jeong, E.‐K. , Desai, K. , & Yurgelun‐Todd, D. (2011). Network anticorrelations, global regression, and phase‐shifted soft tissue correction. Human Brain Mapping, 32(6), 919–934. 10.1002/hbm.21079 20533557 PMC3220164

[hbm26751-bib-0004] Arias, A. J. , Ma, L. , Bjork, J. M. , Hammond, C. J. , Zhou, Y. , Snyder, A. , & Moeller, F. G. (2021). Altered effective connectivity of the reward network during an incentive‐processing task in adults with alcohol use disorder. Alcoholism: Clinical and Experimental Research, 45(8), 1563–1577. 10.1111/acer.14650 34120362 PMC8742221

[hbm26751-bib-0005] Avants, B. B. , Epstein, C. L. , Grossman, M. , & Gee, J. C. (2008). Symmetric diffeomorphic image registration with cross‐correlation: Evaluating automated labeling of elderly and neurodegenerative brain. Medical Image Analysis, 12(1), 26–41. 10.1016/j.media.2007.06.004 17659998 PMC2276735

[hbm26751-bib-0006] Beheshtian, E. , Jalilianhasanpour, R. , Modir Shanechi, A. , Sethi, V. , Wang, G. , Lindquist, M. A. , Caffo, B. S. , Agarwal, S. , Pillai, J. J. , Gujar, S. K. , & Sair, H. I. (2021). Identification of the somatomotor network from language task–based fMRI compared with resting‐state fMRI in patients with brain lesions. Radiology, 301(1), 178–184. 10.1148/radiol.2021204594 34282966

[hbm26751-bib-0007] Bernstein‐Eliav, M. , & Tavor, I. (2024). The prediction of brain activity from connectivity: Advances and applications. The Neuroscientist, 30(3), 367–377. 10.1177/10738584221130974 PMC11107130 36250457

[hbm26751-bib-0008] Biswal, B. B. , Mennes, M. , Zuo, X.‐N. , Gohel, S. , Kelly, C. , Smith Steve, M. , Colcombe, S. , Dogonowski, A.‐M. , Ernst, M. , Fair, D. , Hampson, M. , Hoptman, M. J. , Hyde, J. S. , Kiviniemi, V. J. , Kötter, R. , Li, S.‐J. , … Milham, M. P. (2010). Toward discovery science of human brain function. Proceedings of the National Academy of Sciences, 107(10), 4734–4739. 10.1073/pnas.0911855107 PMC2842060 20176931

[hbm26751-bib-0009] Bossier, H. , Roels, S. P. , Seurinck, R. , Banaschewski, T. , Barker, G. J. , Bokde, A. L. W. , Quinlan, E. B. , Desrivieres, S. , Flor, H. , Gridis, A. , Garavan, H. , Gowland, R. , Heinz, A. , Ittermann, B. , Martinot, J.‐L. , Artiges, E. , Nees, F. , Papadopoulos Orfanos, D. , Poustka, L. , … Moerkerke, B. (2020). The empirical replicability of task‐based fMRI as a function of sample size. NeuroImage, 212, 116601. 10.1016/j.neuroimage.2020.116601 32036019

[hbm26751-bib-0010] Botvinik‐Nezer, R. , Holzmeister, F. , Camerer, C. F. , Dreber, A. , Huber, J. , Johannesson, M. , Kirchler, M. , Iwanir, R. , Mumford, J. A. , Adcock, R. A. , Avesani, P. , Baczkowski, B. M. , Bajracharya, A. , Bakst, L. , Ball, S. , Ball, M. , Bault, N. , Beaton, D. , Beitner, J. , … Schonberg, T. (2020). Variability in the analysis of a single neuroimaging dataset by many teams. Nature, 582(7810), 84–88. 10.1038/s41586-020-2314-9 32483374 PMC7771346

[hbm26751-bib-0011] Boudrias, M.‐H. , Gonçalves, C. S. , Penny, W. D. , Park, C.‐h. , Rossiter, H. E. , Talelli, P. , & Ward, N. S. (2012). Age‐related changes in causal interactions between cortical motor regions during hand grip. NeuroImage, 59(4), 3398–3405. 10.1016/j.neuroimage.2011.11.025 22119651 PMC3315004

[hbm26751-bib-0012] Bühler, M. , Vollstädt‐Klein, S. , Klemen, J. , & Smolka, M. N. (2008). Does erotic stimulus presentation design affect brain activation patterns? Event‐related vs. blocked fMRI designs. Behavioral and Brain Functions, 4(1), 30. 10.1186/1744-9081-4-30 18647397 PMC2515325

[hbm26751-bib-0013] Button, K. S. , Ioannidis, J. P. A. , Mokrysz, C. , Nosek, B. A. , Flint, J. , Robinson, E. S. J. , & Munafò, M. R. (2013). Power failure: Why small sample size undermines the reliability of neuroscience. Nature Reviews Neuroscience, 14(5), 365–376. 10.1038/nrn3475 23571845

[hbm26751-bib-0014] Buxton, R. B. , Uludağ, K. , Dubowitz, D. J. , & Liu, T. T. (2004). Modeling the hemodynamic response to brain activation. NeuroImage, 23, S220–S233. 10.1016/j.neuroimage.2004.07.013 15501093

[hbm26751-bib-0015] Carp, J. (2012). On the plurality of (methodological) worlds: Estimating the analytic flexibility of fMRI experiments. Frontiers in Neuroscience, 6, 33928–33940. 10.3389/fnins.2012.00149 PMC3468892 23087605

[hbm26751-bib-0016] Caspers, S. , Moebus, S. , Lux, S. , Pundt, N. , Schütz, H. , Mühleisen, T. W. , Gras, V. , Eickhoff, S. B. , Romanzetti, S. , Stöcker, T. , Stirnberg, R. , Kirlangic, M. E. , Minnerop, M. , Pieperhoff, P. , Mödder, U. , Das, S. , Evans, A. C. , Jöckel, K.‐H. , Erbel, R. , … Amunts, K. (2014). Studying variability in human brain aging in a population‐based German cohort—Rationale and design of 1000BRAINS. Frontiers in Aging Neuroscience, 6, 149–162. 10.3389/fnagi.2014.00149 25071558 PMC4094912

[hbm26751-bib-0017] Chang, C. , Cunningham, J. P. , & Glover, G. H. (2009). Influence of heart rate on the BOLD signal: The cardiac response function. NeuroImage, 44(3), 857–869. 10.1016/j.neuroimage.2008.09.029 18951982 PMC2677820

[hbm26751-bib-0018] Churchill, N. W. , Spring, R. , Afshin‐Pour, B. , Dong, F. , & Strother, S. C. (2015). An automated, adaptive framework for optimizing preprocessing pipelines in task‐based functional MRI. PLoS One, 10(7), e0131520. 10.1371/journal.pone.0131520 26161667 PMC4498698

[hbm26751-bib-0019] Cieslik, E. C. , Zilles, K. , Grefkes, C. , & Eickhoff, S. B. (2011). Dynamic interactions in the fronto‐parietal network during a manual stimulus–response compatibility task. NeuroImage, 58(3), 860–869. 10.1016/j.neuroimage.2011.05.089 21708271 PMC7998039

[hbm26751-bib-0020] Cieslik, E. C. , Zilles, K. , Kurth, F. , & Eickhoff, S. B. (2010). Dissociating bottom‐up and top‐down processes in a manual stimulus‐response compatibility task. Journal of Neurophysiology, 104(3), 1472–1483. 10.1152/jn.00261.2010 20573974 PMC2944686

[hbm26751-bib-0021] Cole, D. , Smith, S. , & Beckmann, C. (2010). Advances and pitfalls in the analysis and interpretation of resting‐state FMRI data. Frontiers in Systems Neuroscience, 4, 1459–1473. 10.3389/fnsys.2010.00008 PMC2854531 20407579

[hbm26751-bib-0022] Cole, M. W. , Ito, T. , Bassett, D. S. , & Schultz, D. H. (2016). Activity flow over resting‐state networks shapes cognitive task activations. Nature Neuroscience, 19(12), 1718–1726. 10.1038/nn.4406 27723746 PMC5127712

[hbm26751-bib-0023] Cole, M. W. , Bassett, D. S. , Power, J. D. , Braver, T. S. , & Petersen, S. E. (2014). Intrinsic and task‐evoked network architectures of the human brain. Neuron, 83(1), 238–251. 10.1016/j.neuron.2014.05.014 24991964 PMC4082806

[hbm26751-bib-0024] Cox, R. W. (1996). AFNI: Software for analysis and visualization of functional magnetic resonance neuroimages. Computers and Biomedical Research, 29(3), 162–173. 10.1006/cbmr.1996.0014 8812068

[hbm26751-bib-0025] Daunizeau, J. , Preuschoff, K. , Friston, K. , & Stephan, K. (2011). Optimizing experimental design for comparing models of brain function. PLoS Computational Biology, 7(11), e1002280. 10.1371/journal.pcbi.1002280 22125485 PMC3219623

[hbm26751-bib-0026] Davey, C. E. , Grayden, D. B. , Egan, G. F. , & Johnston, L. A. (2013). Filtering induces correlation in fMRI resting state data. NeuroImage, 64, 728–740. 10.1016/j.neuroimage.2012.08.022 22939874

[hbm26751-bib-0027] Desmond, J. E. , & Glover, G. H. (2002). Estimating sample size in functional MRI (fMRI) neuroimaging studies: Statistical power analyses. Journal of Neuroscience Methods, 118(2), 115–128. 10.1016/S0165-0270(02)00121-8 12204303

[hbm26751-bib-0028] Dosenbach, N. U. F. , Visscher, K. M. , Palmer, E. D. , Miezin, F. M. , Wenger, K. K. , Kang, H. C. , Burgund, E. D. , Grimes, A. L. , Schlaggar, B. L. , & Petersen, S. E. (2006). A core system for the implementation of task sets. Neuron, 50(5), 799–812. 10.1016/j.neuron.2006.04.031 16731517 PMC3621133

[hbm26751-bib-0029] Eickhoff, S. B. , Yeo, B. T. T. , & Genon, S. (2018). Imaging‐based parcellations of the human brain. Nature Reviews Neuroscience, 19(11), 672–686. 10.1038/s41583-018-0071-7 30305712

[hbm26751-bib-0030] Esteban, O. , Markiewicz, C. J. , Blair, R. W. , Moodie, C. A. , Isik, A. I. , Erramuzpe, A. , Kent, J. D. , Goncalves, M. , DuPre, E. , Snyder, M. , Oya, H. , Ghosh, S. S. , Wright, J. , Durnez, J. , Poldrack, R. A. , & Gorgolewski, K. J. (2019). fMRIPrep: A robust preprocessing pipeline for functional MRI. Nature Methods, 16(1), 111–116. 10.1038/s41592-018-0235-4 30532080 PMC6319393

[hbm26751-bib-0031] Fitts, P. M. , & Deininger, R. L. (1954). S‐R compatibility: Correspondence among paired elements within stimulus and response codes. Journal of Experimental Psychology, 48(6), 483–492. 10.1037/h0054967 13221745

[hbm26751-bib-0032] Fox, M. D. , Zhang, D. , Snyder, A. Z. , & Raichle, M. E. (2009). The global signal and observed anticorrelated resting state brain networks. Journal of Neurophysiology, 101(6), 3270–3283. 10.1152/jn.90777.2008 19339462 PMC2694109

[hbm26751-bib-0033] Frässle, S. , Lomakina, E. I. , Kasper, L. , Manjaly, Z. M. , Leff, A. , Pruessmann, K. P. , Buhmann, J. M. , & Stephan, K. E. (2018). A generative model of whole‐brain effective connectivity. NeuroImage, 179, 505–529. 10.1016/j.neuroimage.2018.05.058 29807151

[hbm26751-bib-0034] Frässle, S. , Lomakina, E. I. , Razi, A. , Friston, K. J. , Buhmann, J. M. , & Stephan, K. E. (2017). Regression DCM for fMRI. NeuroImage, 155, 406–421. 10.1016/j.neuroimage.2017.02.090 28259780

[hbm26751-bib-0035] Friston, K. J. (2011). Functional and effective connectivity: A review. Brain Connectivity, 1(1), 13–36. 10.1089/brain.2011.0008 22432952

[hbm26751-bib-0036] Friston, K. J. , Harrison, L. , & Penny, W. (2003). Dynamic causal modelling. NeuroImage, 19(4), 1273–1302. 10.1016/S1053-8119(03)00202-7 12948688

[hbm26751-bib-0037] Friston, K. J. , Josephs, O. , Zarahn, E. , Holmes, A. P. , Rouquette, S. , & Poline, J. B. (2000). To smooth or not to smooth?: Bias and efficiency in fMRI time‐series analysis. NeuroImage, 12(2), 196–208. 10.1006/nimg.2000.0609 10913325

[hbm26751-bib-0038] Friston, K. J. , Kahan, J. , Biswal, B. , & Razi, A. (2014). A DCM for resting state fMRI. NeuroImage, 94, 396–407. 10.1016/j.neuroimage.2013.12.009 24345387 PMC4073651

[hbm26751-bib-0039] Friston, K. J. , Litvak, V. , Oswal, A. , Razi, A. , Stephan, K. E. , van Wijk, B. C. M. , Ziegler, G., & Zeidman, P. (2016). Bayesian model reduction and empirical Bayes for group (DCM) studies. NeuroImage, 128, 413–431. 10.1016/j.neuroimage.2015.11.015 26569570 PMC4767224

[hbm26751-bib-0040] Friston, K. J. , Williams, S. , Howard, R. , Frackowiak, R. S. J. , & Turner, R. (1996). Movement‐related effects in fMRI time‐series. Magnetic Resonance in Medicine, 35(3), 346–355. 10.1002/mrm.1910350312 8699946

[hbm26751-bib-0041] Friston, K. J. , Zarahn, E. , Josephs, O. , Henson, R. N. A. , & Dale, A. M. (1999). Stochastic designs in event‐related fMRI. NeuroImage, 10(5), 607–619. 10.1006/nimg.1999.0498 10547338

[hbm26751-bib-0042] Glasser, M. F. , Coalson, T. S. , Bijsterbosch, J. D. , Harrison, S. J. , Harms, M. P. , Anticevic, A. , Van Essen, D. C., & Smith, S. M. (2018). Using temporal ICA to selectively remove global noise while preserving global signal in functional MRI data. NeuroImage, 181, 692–717. 10.1016/j.neuroimage.2018.04.076 29753843 PMC6237431

[hbm26751-bib-0043] Glasser, M. F. , Sotiropoulos, S. N. , Wilson, J. A. , Coalson, T. S. , Fischl, B. , Andersson, J. L. , Xu, J. , Jbabdi, S. , Webster, M. , Polimeni, J. R. , Van Essen, D. C. , & Jenkinson, M. (2013). The minimal preprocessing pipelines for the Human Connectome Project. NeuroImage, 80, 105–124. 10.1016/j.neuroimage.2013.04.127 23668970 PMC3720813

[hbm26751-bib-0044] Goulden, N. , Elliott, R. , Suckling, J. , Williams, S. R. , Deakin, J. F. W. , & McKie, S. (2012). Sample size estimation for comparing parameters using dynamic causal modeling. Brain Connectivity, 2(2), 80–90. 10.1089/brain.2011.0057 22559836

[hbm26751-bib-0045] Greene, A. S. , Gao, S. , Scheinost, D. , & Constable, R. T. (2018). Task‐induced brain state manipulation improves prediction of individual traits. Nature Communications, 9(1), 2807. 10.1038/s41467-018-04920-3 PMC6052101 30022026

[hbm26751-bib-0046] Guo, Q. , Thabane, L. , Hall, G. , McKinnon, M. , Goeree, R. , & Pullenayegum, E. (2014). A systematic review of the reporting of sample size calculations and corresponding data components in observational functional magnetic resonance imaging studies. NeuroImage, 86, 172–181. 10.1016/j.neuroimage.2013.08.012 23954487

[hbm26751-bib-0047] Heckner, M. K. , Cieslik, E. C. , Küppers, V. , Fox, P. T. , Eickhoff, S. B. , & Langner, R. (2021). Delineating visual, auditory and motor regions in the human brain with functional neuroimaging: A BrainMap‐based meta‐analytic synthesis. Scientific Reports, 11(1), 9942. 10.1038/s41598-021-88773-9 33976234 PMC8113600

[hbm26751-bib-0048] Hofmann, D. , & Straube, T. (2019). Resting‐state fMRI effective connectivity between the bed nucleus of the stria terminalis and amygdala nuclei. Human Brain Mapping, 40(9), 2723–2735. 10.1002/hbm.24555 30829454 PMC6865382

[hbm26751-bib-0049] Huettel, S. A. (2012). Event‐related fMRI in cognition. NeuroImage, 62(2), 1152–1156. 10.1016/j.neuroimage.2011.08.113 21963919 PMC3277683

[hbm26751-bib-0050] Jenkinson, M. , Bannister, P. , Brady, M. , & Smith, S. (2002). Improved optimization for the robust and accurate linear registration and motion correction of brain images. NeuroImage, 17(2), 825–841. 10.1006/nimg.2002.1132 12377157

[hbm26751-bib-0051] Jenkinson, M. , Beckmann, C. F. , Behrens, T. E. J. , Woolrich, M. W. , & Smith, S. M. (2012). Fsl. Neuroimage, 62(2), 782–790. 10.1016/j.neuroimage.2011.09.015 21979382

[hbm26751-bib-0052] Jülich Supercomputing Centre . (2021). JURECA: Data centric and booster modules implementing the modular supercomputing architecture at Jülich Supercomputing Centre. Journal of Large‐Scale Research Facilities, 7, 182–190. 10.17815/jlsrf-7-182

[hbm26751-bib-0053] Jung, K. , Friston, K. J. , Pae, C. , Choi, H. H. , Tak, S. , Choi, Y. K. , Park, B. , Park, C.‐A. , Cheong, C. , & Park, H.‐J. (2018). Effective connectivity during working memory and resting states: A DCM study. NeuroImage, 169, 485–495. 10.1016/j.neuroimage.2017.12.067 29284140

[hbm26751-bib-0054] Kahan, J. , & Foltynie, T. (2013). Understanding DCM: Ten simple rules for the clinician. NeuroImage, 83, 542–549. 10.1016/j.neuroimage.2013.07.008 23850463

[hbm26751-bib-0055] Kahan, J. , Mancini, L. , Flandin, G. , White, M. , Papadaki, A. , Thornton, J. , Yousry, T. , Zrinzo, L. , Hariz, M. , Limousin, P. , Friston, K. , & Foltynie, T. (2019). Deep brain stimulation has state‐dependent effects on motor connectivity in Parkinson's disease. Brain, 142(8), 2417–2431. 10.1093/brain/awz164 31219504 PMC7053573

[hbm26751-bib-0056] Kuhnke, P. , Kiefer, M. , & Hartwigsen, G. (2021). Task‐dependent functional and effective connectivity during conceptual processing. Cerebral Cortex, 31(7), 3475–3493. 10.1093/cercor/bhab026 33677479 PMC8196308

[hbm26751-bib-0057] Langner, R. , Cieslik, E. C. , Behrwind, S. D. , Roski, C. , Caspers, S. , Amunts, K. , & Eickhoff, S. B. (2015). Aging and response conflict solution: Behavioural and functional connectivity changes. Brain Structure and Function, 220(3), 1739–1757. 10.1007/s00429-014-0758-0 24718622 PMC4193951

[hbm26751-bib-0058] Liu, T. T. , Frank, L. R. , Wong, E. C. , & Buxton, R. B. (2001). Detection power, estimation efficiency, and predictability in event‐related fMRI. NeuroImage, 13(4), 759–773. 10.1006/nimg.2000.0728 11305903

[hbm26751-bib-0059] Liu, T. T. , Nalci, A. , & Falahpour, M. (2017). The global signal in fMRI: Nuisance or information? NeuroImage, 150, 213–229. 10.1016/j.neuroimage.2017.02.036 28213118 PMC5406229

[hbm26751-bib-0060] Loehrer, P. A. , Nettersheim, F. S. , Jung, F. , Weber, I. , Huber, C. , Dembek, T. A. , Pelzer, E. A. , Fink, G. R. , Tittgemeyer, M. , & Timmermann, L. (2016). Ageing changes effective connectivity of motor networks during bimanual finger coordination. NeuroImage, 143, 325–342. 10.1016/j.neuroimage.2016.09.014 27616642

[hbm26751-bib-0061] Logothetis, N. K. (2008). What we can do and what we cannot do with fMRI. Nature, 453(7197), 869–878. 10.1038/nature06976 18548064

[hbm26751-bib-0062] Ma, L. , Steinberg, J. L. , Cunningham, K. A. , Lane, S. D. , Kramer, L. A. , Narayana, P. A. , Kosten, T. R. , Bechara, A. , & Moeller, F. G. (2014). Inhibitory behavioral control: A stochastic dynamic causal modeling study using network discovery analysis. Brain Connectivity, 5(3), 177–186. 10.1089/brain.2014.0275 25336321 PMC4394161

[hbm26751-bib-0063] Mascali, D. , Moraschi, M. , DiNuzzo, M. , Tommasin, S. , Fratini, M. , Gili, T. , Wise, R. G. , Mangia, S. , Macaluso, E. , & Giove, F. (2021). Evaluation of denoising strategies for task‐based functional connectivity: Equalizing residual motion artifacts between rest and cognitively demanding tasks. Human Brain Mapping, 42(6), 1805–1828. 10.1002/hbm.25332 33528884 PMC7978116

[hbm26751-bib-0064] Mechelli, A. , Henson, R. N. A. , Price, C. J. , & Friston, K. J. (2003). Comparing event‐related and epoch analysis in blocked design fMRI. NeuroImage, 18(3), 806–810. 10.1016/S1053-8119(02)00027-7 12667857

[hbm26751-bib-0065] Mechelli, A. , Price, C. J. , Henson, R. N. A. , & Friston, K. J. (2003). Estimating efficiency a priori: A comparison of blocked and randomized designs. NeuroImage, 18(3), 798–805. 10.1016/S1053-8119(02)00040-X 12667856

[hbm26751-bib-0066] Menon, V. (2011). Large‐scale brain networks and psychopathology: A unifying triple network model. Trends in Cognitive Sciences, 15(10), 483–506. 10.1016/j.tics.2011.08.003 21908230

[hbm26751-bib-0067] Menon, V. , & Uddin, L. Q. (2010). Saliency, switching, attention and control: A network model of insula function. Brain Structure and Function, 214(5), 655–667. 10.1007/s00429-010-0262-0 20512370 PMC2899886

[hbm26751-bib-0068] Morken, F. , Helland, T. , Hugdahl, K. , & Specht, K. (2017). Reading in dyslexia across literacy development: A longitudinal study of effective connectivity. NeuroImage, 144, 92–100. 10.1016/j.neuroimage.2016.09.060 27688204

[hbm26751-bib-0069] Munoz, D. P. , & Everling, S. (2004). Look away: The anti‐saccade task and the voluntary control of eye movement. Nature Reviews Neuroscience, 5(3), 218–228. 10.1038/nrn1345 14976521

[hbm26751-bib-0070] Murphy, K. , Birn, R. M. , Handwerker, D. A. , Jones, T. B. , & Bandettini, P. A. (2009). The impact of global signal regression on resting state correlations: Are anti‐correlated networks introduced? NeuroImage, 44(3), 893–905. 10.1016/j.neuroimage.2008.09.036 18976716 PMC2750906

[hbm26751-bib-0071] Murphy, K. , & Fox, M. D. (2017). Towards a consensus regarding global signal regression for resting state functional connectivity MRI. NeuroImage, 154, 169–173. 10.1016/j.neuroimage.2016.11.052 27888059 PMC5489207

[hbm26751-bib-0072] Nee, D. E. , Wager, T. D. , & Jonides, J. (2007). Interference resolution: Insights from a meta‐analysis of neuroimaging tasks. Cognitive, Affective, & Behavioral Neuroscience, 7(1), 1–17. 10.3758/CABN.7.1.1 17598730

[hbm26751-bib-0073] Park, H.‐J. , Friston, K. J. , Pae, C. , Park, B. , & Razi, A. (2018). Dynamic effective connectivity in resting state fMRI. NeuroImage, 180, 594–608. 10.1016/j.neuroimage.2017.11.033 29158202 PMC6138953

[hbm26751-bib-0074] Parker, D. B. , & Razlighi, Q. R. (2019). The benefit of slice timing correction in common fMRI preprocessing pipelines. Frontiers in Neuroscience, 13, 465275–465296. 10.3389/fnins.2019.00821 PMC6736626 31551667

[hbm26751-bib-0075] Petersen, S. E. , & Dubis, J. W. (2012). The mixed block/event‐related design. NeuroImage, 62(2), 1177–1184. 10.1016/j.neuroimage.2011.09.084 22008373 PMC3288695

[hbm26751-bib-0076] Power, J. D. , Mitra, A. , Laumann, T. O. , Snyder, A. Z. , Schlaggar, B. L. , & Petersen, S. E. (2014). Methods to detect, characterize, and remove motion artifact in resting state fMRI. NeuroImage, 84, 320–341. 10.1016/j.neuroimage.2013.08.048 23994314 PMC3849338

[hbm26751-bib-0077] Power, J. D. , Plitt, M. , Laumann, T. O. , & Martin, A. (2017). Sources and implications of whole‐brain fMRI signals in humans. NeuroImage, 146, 609–625. 10.1016/j.neuroimage.2016.09.038 27751941 PMC5321814

[hbm26751-bib-0078] Reuter‐Lorenz, P. A. , & Park, D. C. (2010). Human neuroscience and the aging mind: A new look at old problems. The Journals of Gerontology: Series B, 65B(4), 405–415. 10.1093/geronb/gbq035 PMC2883872 20478901

[hbm26751-bib-0079] Roels, S. P. , Bossier, H. , Loeys, T. , & Moerkerke, B. (2015). Data‐analytical stability of cluster‐wise and peak‐wise inference in fMRI data analysis. Journal of Neuroscience Methods, 240, 37–47. 10.1016/j.jneumeth.2014.10.024 25445059

[hbm26751-bib-0080] Saad, Z. S. , Gotts, S. J. , Murphy, K. , Chen, G. , Jo, H. J. , Martin, A. , & Cox, R. W. (2012). Trouble at rest: How correlation patterns and group differences become distorted after global signal regression. Brain Connectivity, 2(1), 25–32. 10.1089/brain.2012.0080 22432927 PMC3484684

[hbm26751-bib-0081] Shen, X. , Finn, E. S. , Scheinost, D. , Rosenberg, M. D. , Chun, M. M. , Papademetris, X. , & Constable, R. T. (2017). Using connectome‐based predictive modeling to predict individual behavior from brain connectivity. Nature Protocols, 12(3), 506–518. 10.1038/nprot.2016.178 28182017 PMC5526681

[hbm26751-bib-0082] Sladky, R. , Friston, K. J. , Tröstl, J. , Cunnington, R. , Moser, E. , & Windischberger, C. (2011). Slice‐timing effects and their correction in functional MRI. NeuroImage, 58(2), 588–594. 10.1016/j.neuroimage.2011.06.078 21757015 PMC3167249

[hbm26751-bib-0083] Smith, S. M. (2004). Overview of fMRI analysis. The British Journal of Radiology, 77(suppl_2), S167–S175. 10.1259/bjr/33553595 15677358

[hbm26751-bib-0084] Smith, S. M. , & Nichols, T. E. (2009). Threshold‐free cluster enhancement: Addressing problems of smoothing, threshold dependence and localisation in cluster inference. NeuroImage, 44(1), 83–98. 10.1016/j.neuroimage.2008.03.061 18501637

[hbm26751-bib-0085] Smith, S. M. , Vidaurre, D. , Beckmann, C. F. , Glasser, M. F. , Jenkinson, M. , Miller, K. L. , Nichols, T. E. , Robinson, E. C. , Gholamreza, S.‐K. , Woolrich, M. W. , Barch, D. M. , Ugurbil, K. , & Van Essen, D. C. (2013). Functional connectomics from resting‐state fMRI. Trends in Cognitive Sciences, 17(12), 666–682. 10.1016/j.tics.2013.09.016 24238796 PMC4004765

[hbm26751-bib-0086] Stephan, K. E. , Penny, W. D. , Daunizeau, J. , Moran, R. J. , & Friston, K. J. (2009). Bayesian model selection for group studies. NeuroImage, 46(4), 1004–1017. 19306932 10.1016/j.neuroimage.2009.03.025 PMC2703732

[hbm26751-bib-0087] Taha, A. A. , & Hanbury, A. (2015). Metrics for evaluating 3D medical image segmentation: Analysis, selection, and tool. BMC Medical Imaging, 15(1), 29. 10.1186/s12880-015-0068-x 26263899 PMC4533825

[hbm26751-bib-0088] Thirion, B. , Pinel, P. , Mériaux, S. , Roche, A. , Dehaene, S. , & Poline, J.‐B. (2007). Analysis of a large fMRI cohort: Statistical and methodological issues for group analyses. NeuroImage, 35(1), 105–120. 10.1016/j.neuroimage.2006.11.054 17239619

[hbm26751-bib-0089] Tie, Y. , Suarez, R. O. , Whalen, S. , Radmanesh, A. , Norton, I. H. , & Golby, A. J. (2009). Comparison of blocked and event‐related fMRI designs for pre‐surgical language mapping. NeuroImage, 47, T107–T115. 10.1016/j.neuroimage.2008.11.020 19101639 PMC3036974

[hbm26751-bib-0090] Tuominen, J. , Specht, K. , Vaisvilaite, L. , & Zeidman, P. (2023). An information‐theoretic analysis of resting‐state versus task fMRI. Network Neuroscience, 1‐18, 769–786. 10.1162/netn_a_00302 PMC10312267 37397893

[hbm26751-bib-0091] Turner, B. O. , Paul, E. J. , Miller, M. B. , & Barbey, A. K. (2018). Small sample sizes reduce the replicability of task‐based fMRI studies. Communications Biology, 1(1), 62. 10.1038/s42003-018-0073-z 30271944 PMC6123695

[hbm26751-bib-0092] Tustison, N. J. , Avants, B. B. , Cook, P. A. , Zheng, Y. , Egan, A. , Yushkevich, P. A. , & Gee, J. C. (2010). N4ITK: Improved N3 bias correction. IEEE Transactions on Medical Imaging, 29(6), 1310–1320. 10.1109/TMI.2010.2046908 20378467 PMC3071855

[hbm26751-bib-0093] Tustison, N. J. , Cook, P. A. , Klein, A. , Song, G. , Das, S. R. , Duda, J. T. , Kandel, B. M. , van Strien, N. , Stone, J. R. , Gee, J. C. , & Avants, B. B. (2014). Large‐scale evaluation of ANTs and FreeSurfer cortical thickness measurements. NeuroImage, 99, 166–179. 10.1016/j.neuroimage.2014.05.044 24879923

[hbm26751-bib-0094] van den Heuvel, M. P. , & Hulshoff Pol, H. E. (2010). Exploring the brain network: A review on resting‐state fMRI functional connectivity. European Neuropsychopharmacology, 20(8), 519–534. 10.1016/j.euroneuro.2010.03.008 20471808

[hbm26751-bib-0095] Varikuti, D. P. , Hoffstaedter, F. , Genon, S. , Schwender, H. , Reid, A. T. , & Eickhoff, S. B. (2017). Resting‐state test–retest reliability of a priori defined canonical networks over different preprocessing steps. Brain Structure and Function, 222(3), 1447–1468. 10.1007/s00429-016-1286-x 27550015 PMC5322256

[hbm26751-bib-0096] Visscher, K. M. , Miezin, F. M. , Kelly, J. E. , Buckner, R. L. , Donaldson, D. I. , McAvoy, M. P. , Bhalodia, V. M., & Petersen, S. E. (2003). Mixed blocked/event‐related designs separate transient and sustained activity in fMRI. NeuroImage, 19(4), 1694–1708. 10.1016/S1053-8119(03)00178-2 12948724

[hbm26751-bib-0097] Volz, L. J. , Eickhoff, S. B. , Pool, E.‐M. , Fink, G. R. , & Grefkes, C. (2015). Differential modulation of motor network connectivity during movements of the upper and lower limbs. NeuroImage, 119, 44–53. 10.1016/j.neuroimage.2015.05.101 26095089

[hbm26751-bib-0098] Weissenbacher, A. , Kasess, C. , Gerstl, F. , Lanzenberger, R. , Moser, E. , & Windischberger, C. (2009). Correlations and anticorrelations in resting‐state functional connectivity MRI: A quantitative comparison of preprocessing strategies. NeuroImage, 47(4), 1408–1416. 10.1016/j.neuroimage.2009.05.005 19442749

[hbm26751-bib-0099] Winkler, A. M. , Ridgway, G. R. , Webster, M. A. , Smith, S. M. , & Nichols, T. E. (2014). Permutation inference for the general linear model. NeuroImage, 92, 381–397. 10.1016/j.neuroimage.2014.01.060 24530839 PMC4010955

[hbm26751-bib-0100] Woolrich, M. W. , Behrens, T. E. J. , & Smith, S. M. (2004). Constrained linear basis sets for HRF modelling using variational Bayes. NeuroImage, 21(4), 1748–1761. 10.1016/j.neuroimage.2003.12.024 15050595

[hbm26751-bib-0101] Yan, C.‐G. , Craddock, R. C. , He, Y. , & Milham, M. (2013). Addressing head motion dependencies for small‐world topologies in functional connectomics. Frontiers in Human Neuroscience, 7, 910–928. 10.3389/fnhum.2013.00910 24421764 PMC3872728

[hbm26751-bib-0102] Yeo, B. T. , Krienen, F. M. , Sepulcre, J. , Sabuncu, M. R. , Lashkari, D. , Hollinshead, M. , Roffman, J. L., Smoller, J. W., Zöllei, L., Polimeni, J. R., Fischl, B., Liu, H., & Buckner, R. L. (2011). The organization of the human cerebral cortex estimated by intrinsic functional connectivity. Journal of Neurophysiology, 106(3), 1125–1165. 10.1152/jn.00338.2011 21653723 PMC3174820

[hbm26751-bib-0103] Zeidman, P. , Jafarian, A. , Corbin, N. , Seghier, M. L. , Razi, A. , Price, C. J. , & Friston, K. J. (2019). A guide to group effective connectivity analysis, part 1: First level analysis with DCM for fMRI. NeuroImage, 200, 174–190. 10.1016/j.neuroimage.2019.06.031 31226497 PMC6711459

[hbm26751-bib-0104] Zeidman, P. , Jafarian, A. , Seghier, M. L. , Litvak, V. , Cagnan, H. , Price, C. J. , & Friston, K. J. (2019). A guide to group effective connectivity analysis, part 2: Second level analysis with PEB. NeuroImage, 200, 12–25. 10.1016/j.neuroimage.2019.06.032 31226492 PMC6711451

[hbm26751-bib-0105] Zeidman, P. , Kazan, S. M. , Todd, N. , Weiskopf, N. , Friston, K. J. , & Callaghan, M. F. (2019). Optimizing data for modeling neuronal responses. Frontiers in Neuroscience, 12, 411195–411210. 10.3389/fnins.2018.00986 PMC6335328 30686967

[hbm26751-bib-0106] Zhang, Y. , Brady, M. , & Smith, S. (2001). Segmentation of brain MR images through a hidden Markov random field model and the expectation‐maximization algorithm. IEEE Transactions on Medical Imaging, 20(1), 45–57. 11293691 10.1109/42.906424

