# Supplementary material for: Impact of data processing varieties on DCM estimates of effective connectivity from task‐fMRI
Source: Hum Brain Mapp. 2024 Jun 12;45(8):e26751. doi: 10.1002/hbm.26751 (PMC11167406; doi:10.1002/hbm.26751)
Supplement: Supplementary file 1 — DATA S1: Supporting information. [file HBM-45-e26751-s001.docx]

Supplementary materials

for the manuscript entitled

Impact of data processing varieties on DCM estimates of effective connectivity from task-fMRI

by

Shufei Zhang^1,2^, Kyesam Jung^1,2^, Robert Langner^1,2^, Esther Florin^3^, Simon B. Eickhoff^1,2^, Oleksandr V. Popovych^1,2^

^1^Institute of Neuroscience and Medicine, Brain and Behaviour (INM-7), Research Centre Jülich, Germany

^2^Institute for Systems Neuroscience, Medical Faculty, Heinrich-Heine University Düsseldorf, Germany

^3^Institute of Clinical Neuroscience and Medical Psychology, Medical Faculty, Heinrich-Heine University Düsseldorf, Germany

*Corresponding author: o.popovych@fz-juelich.de

## Supplementary Figures


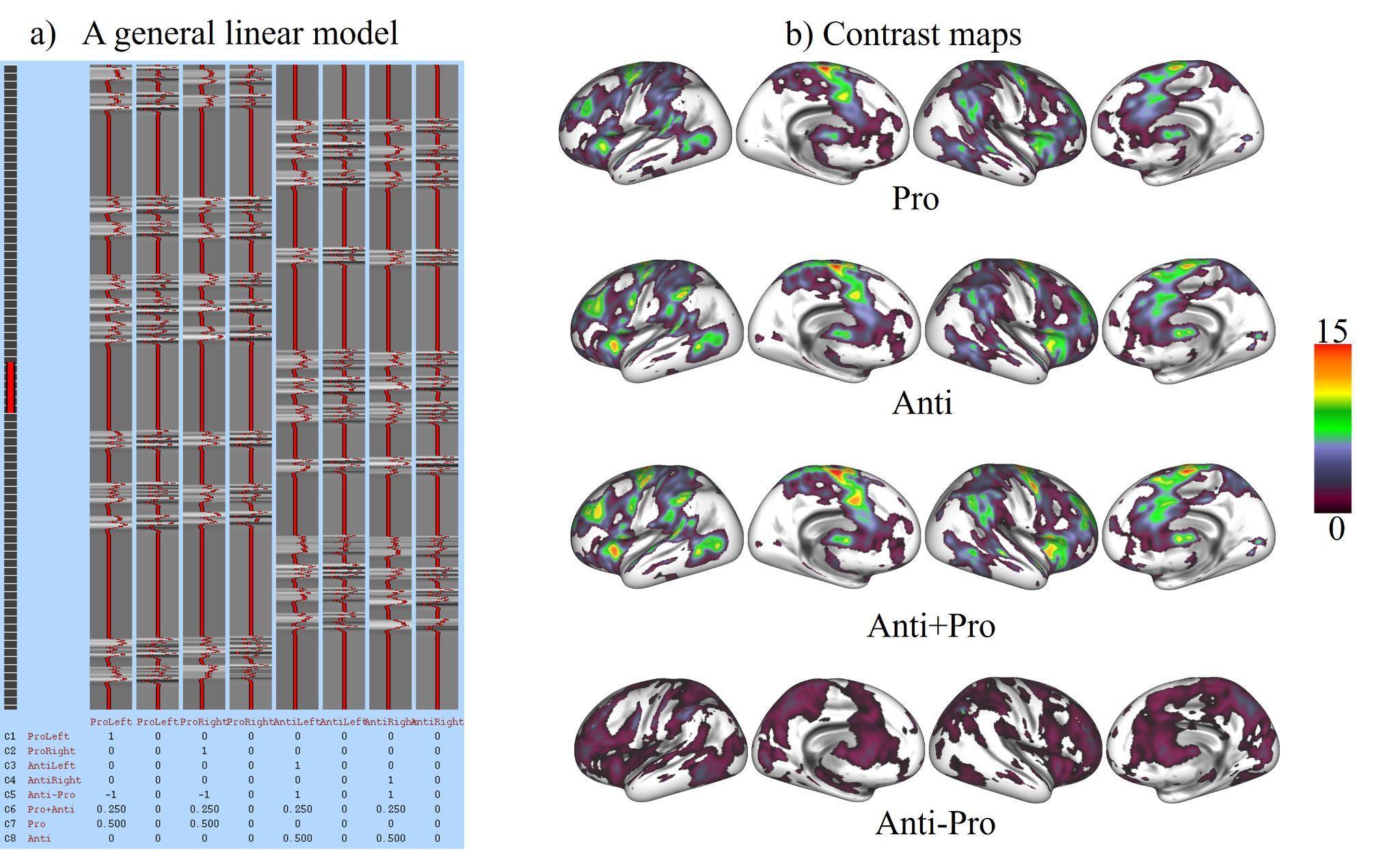


Fig. 1. The case for FSL general linear model (GLM) and contrast maps from the event-related design. A double gamma hemodynamic response function (odd columns) and temporal derivatives (even columns) are presented in the (a). Four contrast maps are shown in (b). The Pro or Anti contrast maps indicated the activated regions under congruent or incongruent conditions. The Anti+Pro contrast map indicated the group-mean activated regions under both conditions. The Anti > Pro contrast map indicated the activated regions associated with the incompatibility effect.


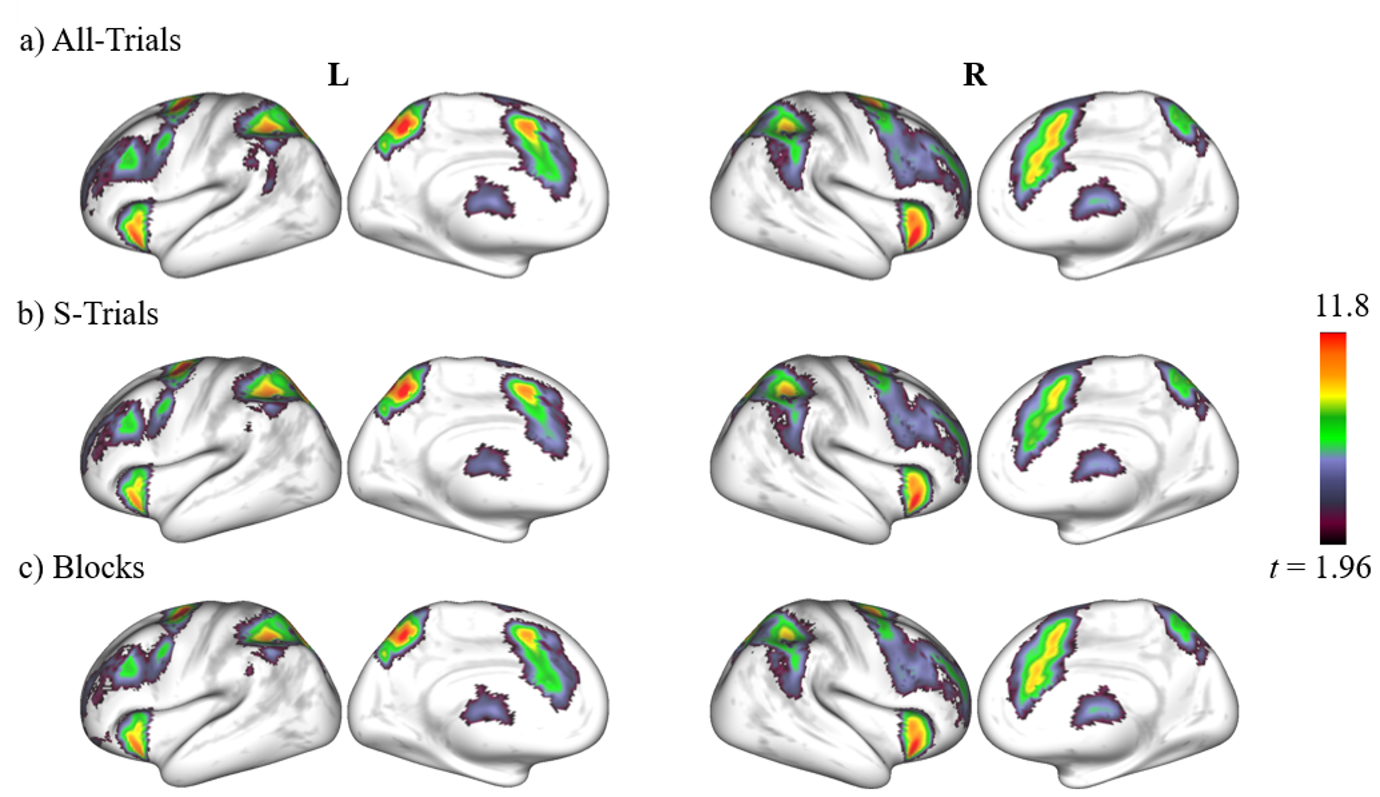


Fig. 2. Results of the second-level fMRI analysis for different GLM designs by considering (a) All-Trials, (b) S-Trials, and (c) Blocks designs without GSR. All maps illustrate the *t*-values (scaling of the color bar) of the t-tests reflecting the statistically significant voxels across all subjects (*p*_TFCE+FWE_ < 0.05, corrected by threshold-free cluster enhancement (TFCE) and family-wise error (FWE) rate methods) of the contrast difference between incongruent and congruent experimental conditions (Anti-Pro contrast). For better visualization, each thresholded statistical map was projected to fs_LR 32k surfaces.


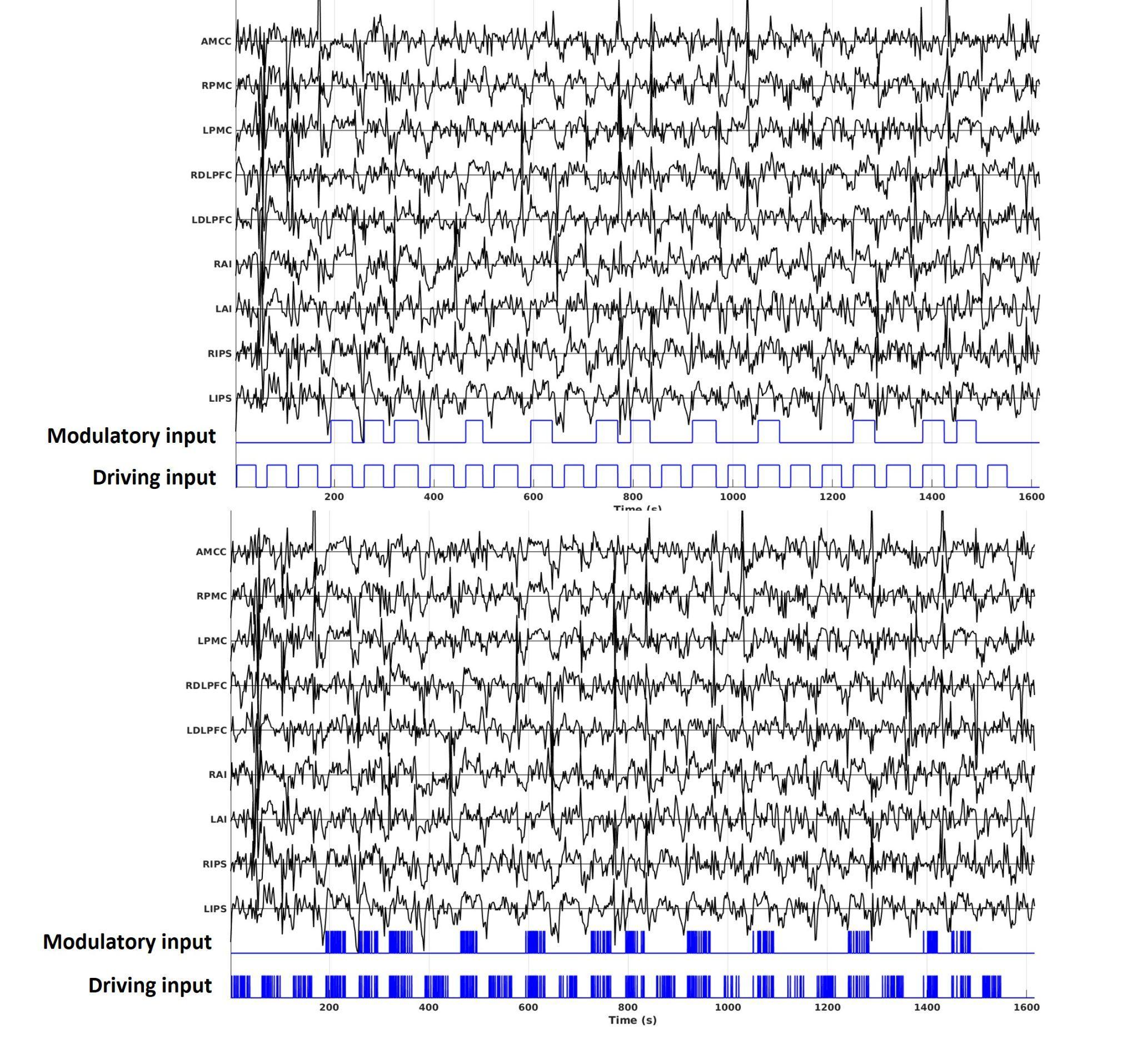


Fig. 3. The example of DCM model specifications from the block design (upper one) and event-related design (lower one) with the Anti-contrast. The first 9 rows illustrate the empirical BOLD signals of 9 nodes involved in the SRC network, while the last two rows indicate the modulatory input (also for the contrast) and driving input (i.e., visual stimuli).


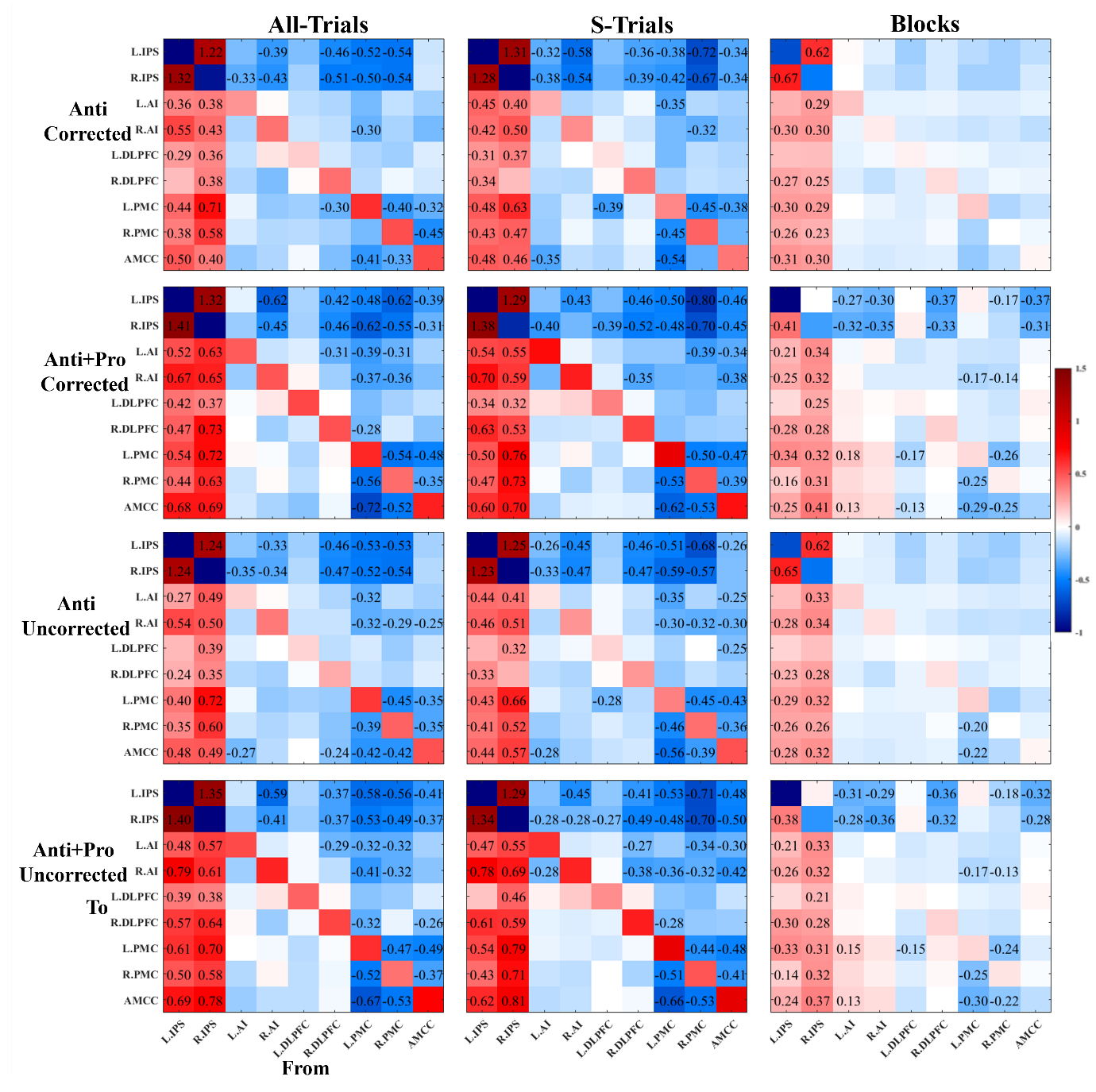


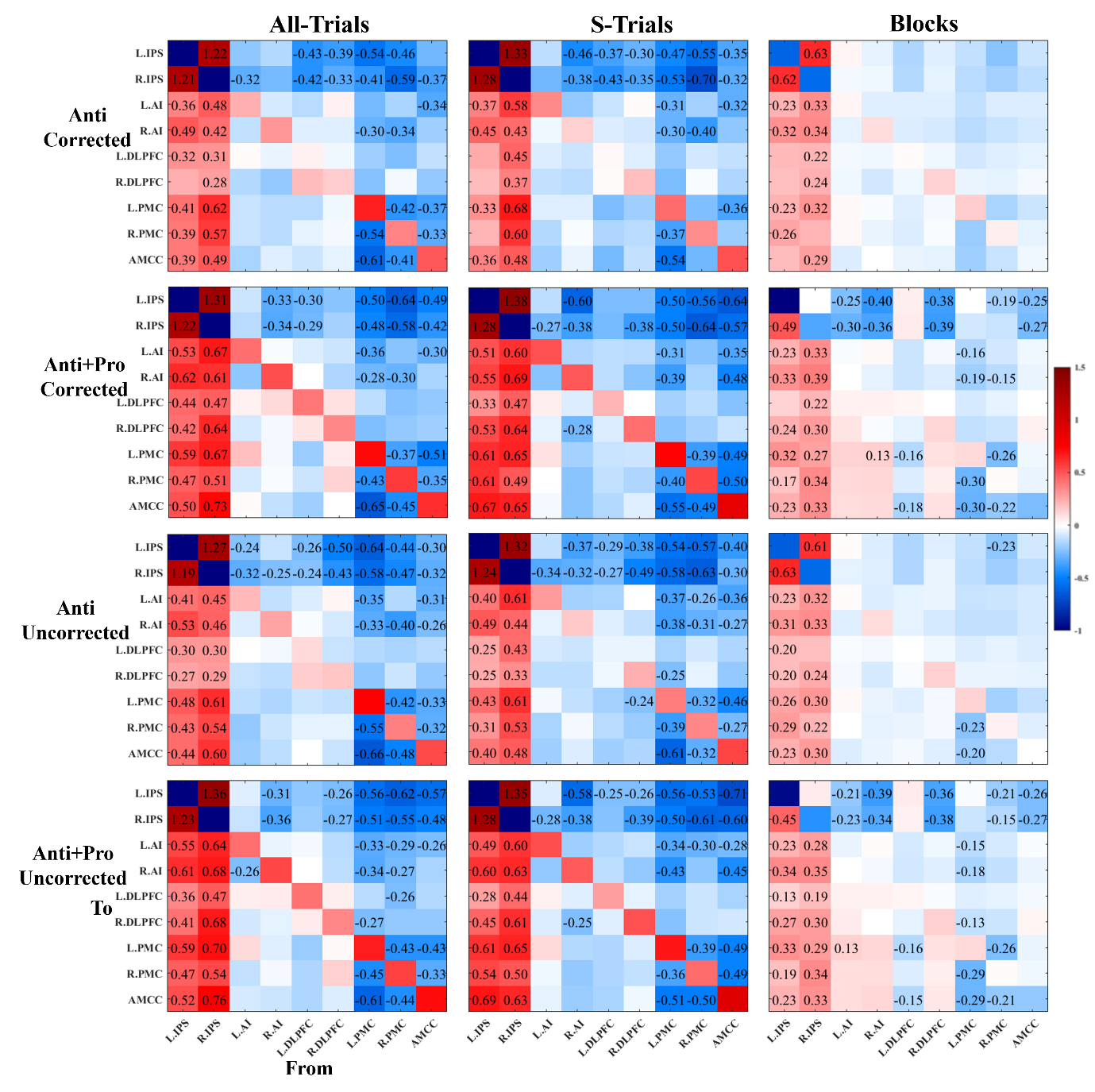


Fig. 4. Group-mean modulatory EC (B matrices) for each considered condition of the data processing indicated on the top and left sides of the plots with GSR (upper one) without GSR (lower one). The color encodes the strength of EC ranging from blue (negative EC) to red (positive EC), where the EC edges above the 95% posterior probability threshold are labeled by the black numbers. The connectivity is directed from the network nodes indicated on the horizontal axes to the network nodes indicated on the vertical axes. See Methods for details and notations.


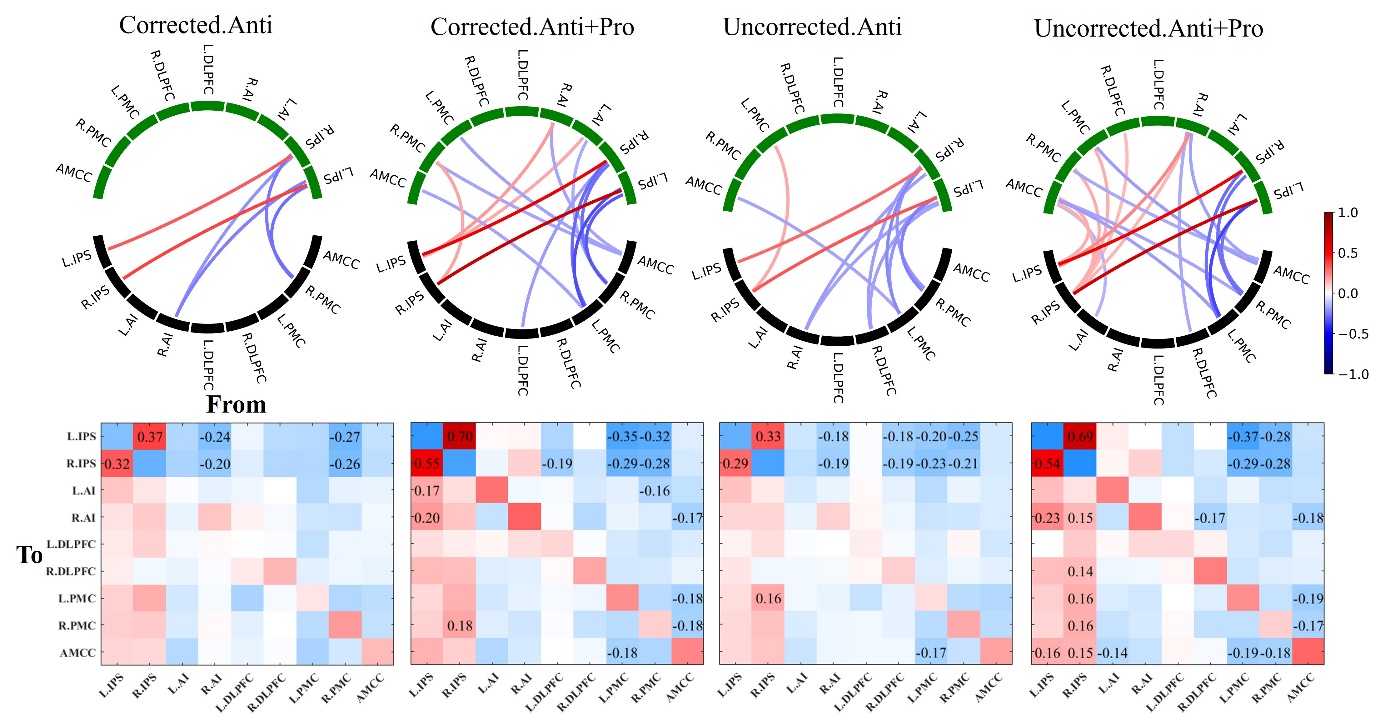


Fig. 5. Comparison of the task-evoked EC (matrix B) between the considered GLM designs (S-Trials vs. Blocks) using between-group PEB analyses. The other considered conditions of the data processing (contrast and thresholding) are indicated in the titles of the plots. In the circular network plots (upper row), the EC edges (exceeding the threshold of PP > 95%) at the group level of the difference S-Trials - Blocks are depicted. The black and green network nodes correspond to the sources (“from”) and destinations (“to”) of the illustrated directed connectivity, respectively. In the matrix plots (lower row), EC differences are also depicted by color, and numbers in the corresponding cells indicate values. The network nodes shown in the horizontal and vertical axes correspond to the sources (“from”) and destinations (“to”) of the directed connectivity, respectively. See Methods of the nodes’ abbreviations.


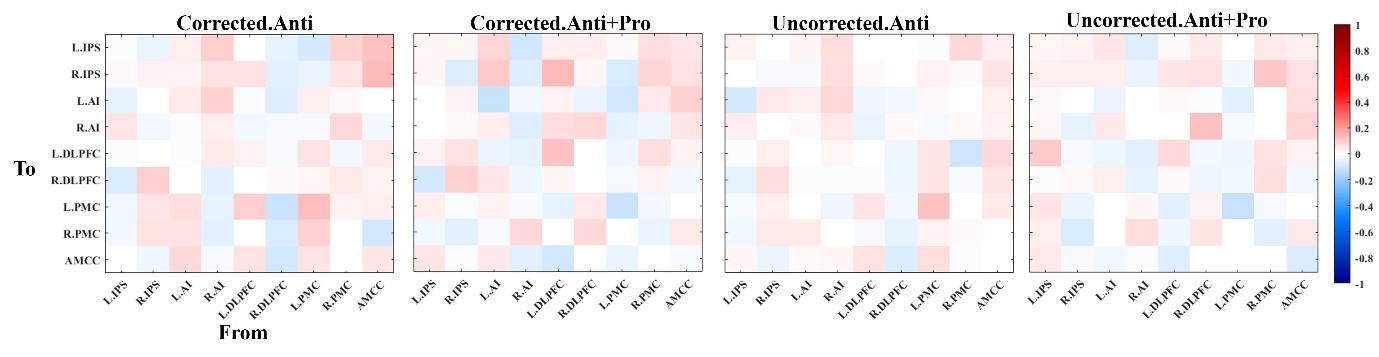


Fig. 6. Comparison of the task-evoked EC (matrix B) between the considered GLM designs (All-Trials vs. S-Trials) using between-group PEB analyses. The other considered conditions of the data processing (contrasts and thresholding) are indicated in the titles of the plots. The values of the connectivity differences are reflected by color as shown in the color bar, but no modulatory EC is above the 95% posterior probability threshold. See Methods of the nodes’ abbreviations.


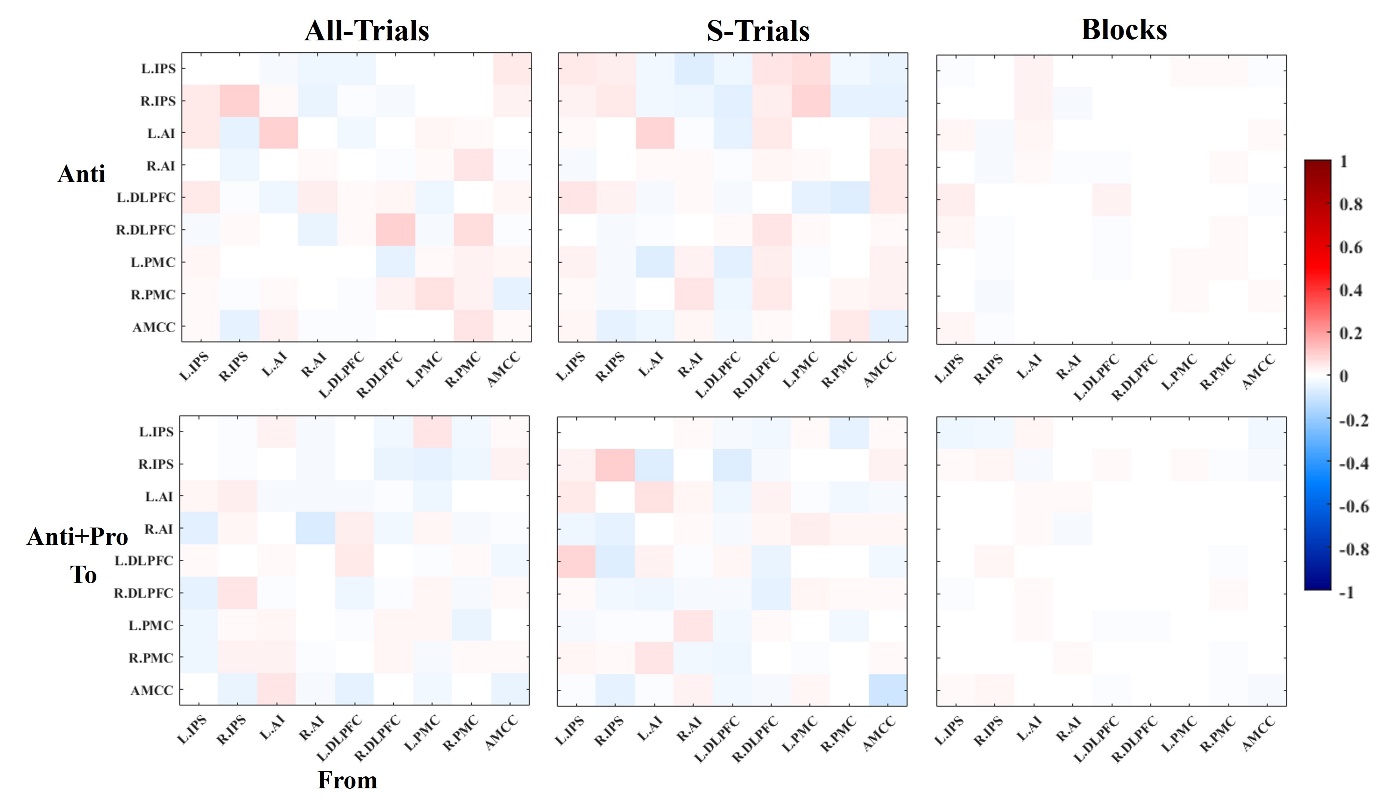


Fig. 7. Comparison of the task-evoked EC (matrix B) between the thresholding conditions (Corrected vs. Uncorrected) using between-group PEB analyses. The other considered conditions of the data processing (contrasts and GLM designs) are indicated in the titles of the plots. The values of the connectivity differences are reflected by color as shown in the color bar, but no modulatory EC is above the 95% posterior probability threshold. See Methods of the nodes’ abbreviations.


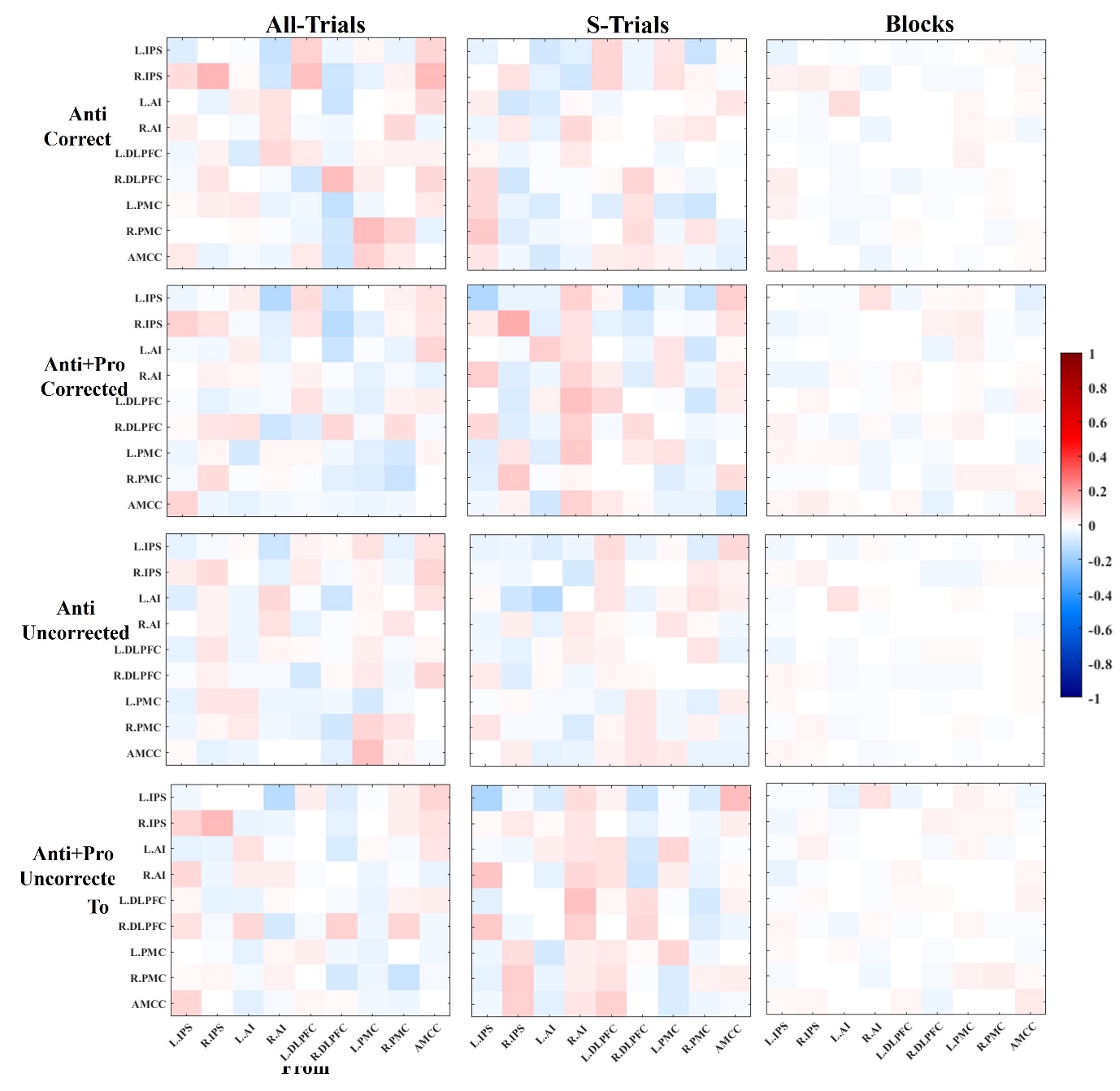


Fig. 8. Comparison of the task-evoked EC (matrix B) between the GSR conditions (With GSR vs. Without GSR). The results of PEB analyses at the group level are illustrated for the differences of B-matrices where the subtraction between conditions with and without GSR is performed: With GSR - Without GSR. The other considered conditions of the data processing (GLM designs, thresholding, and contrasts) are indicated in the titles of the plots. The values of the connectivity differences are reflected by color as indicated in the color bar, but no modulatory EC is above the threshold of 95% posterior probability. See Methods of the nodes’ abbreviations.


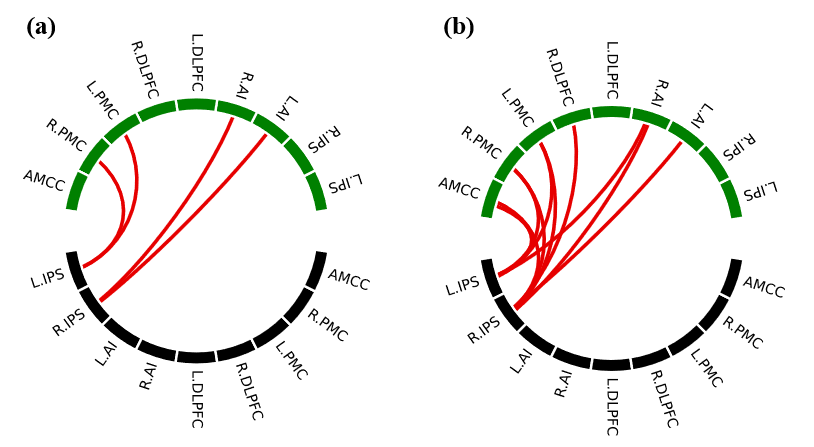


Fig. 9. The stable effective connectivity (EC) edges between group-mean and between-group PEB analyses for cases of (a) All-Trials > Black and (b) Anti+Pro > Anti. The calculation steps included: (1) For simplification, we extracted common EC edges (posterior probability/PP > 95%) from group-mean PEB analyses including the conditions of significance thresholding, All-Trials/Block GLM designs, and activation contrasts. This step offered EC edges that were stable across the conditions above. (2) After common group-mean EC edges (PP > 95%) were specified, we summed up EC edges showing strong evidence between-group PEB differences from all All-Trials > Block and all Anti+Pro > Anti between-group PEB analyses, respectively. (3) The stable EC edges of All-Trials > Block (Fig. 10a) were calculated by removing summed EC edges of All-Trials > Block from the common group-mean EC edges, and the stable EC edges of Anti+Pro > Anti (Fig. 10b) were calculated by removing summed EC edges of Anti+Pro > Anti from the common group-mean EC edges.


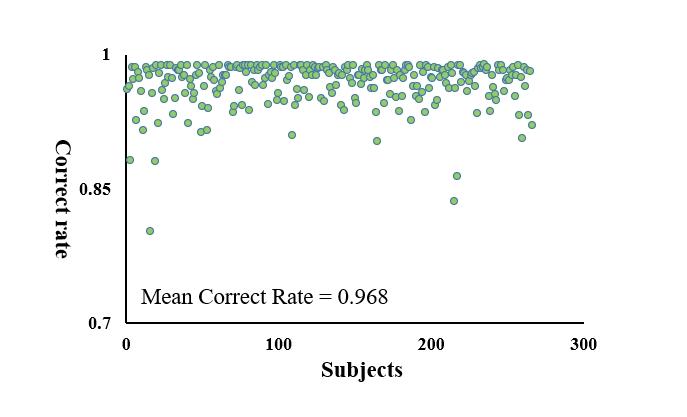


Fig. 10. The correct rate distribution across subjects (n = 266). The green dots indicate subjects, and the mean correct rate is 0.968. The individual correct rate was calculated by 1 - ((erroneous trials + non-response trials) / all trials).


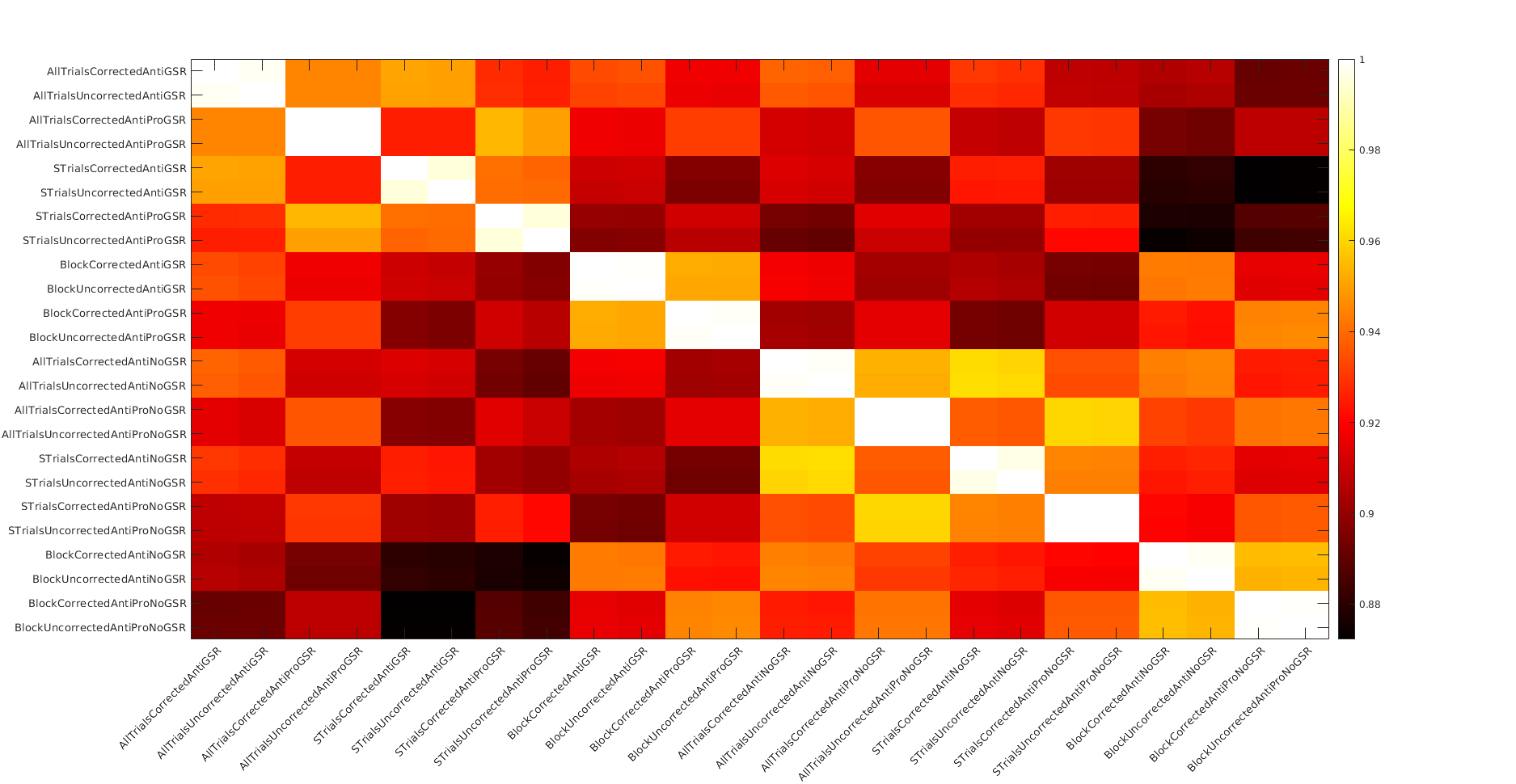


Fig. 11. Cross correlations (*Pearson*) between BOLD time series for any combined pair of considered data-processing conditions indicated on the axes. The time series were extracted for subject-level node ROIs for individual subjects from the same cohort of 90 subjects common (intersection) across all conditions. Node-wise cross-condition correlations were calculated for individual subjects and then averaged across nodes and subjects into one correlation value depicted in color.

| 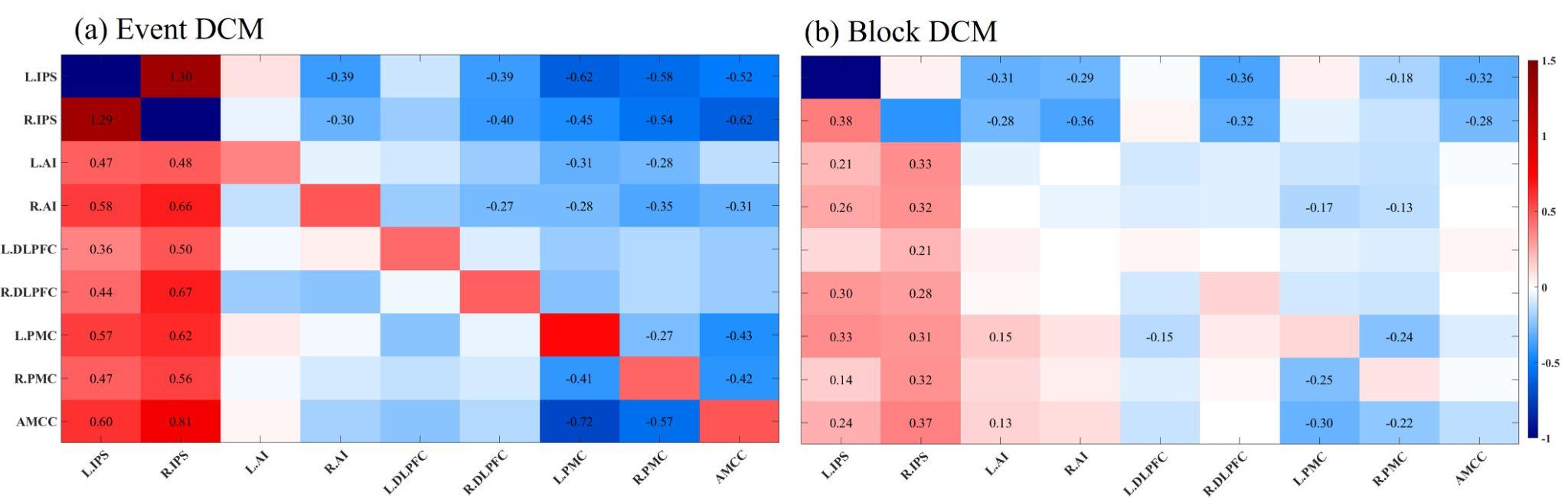 | |
| --- | --- |
| **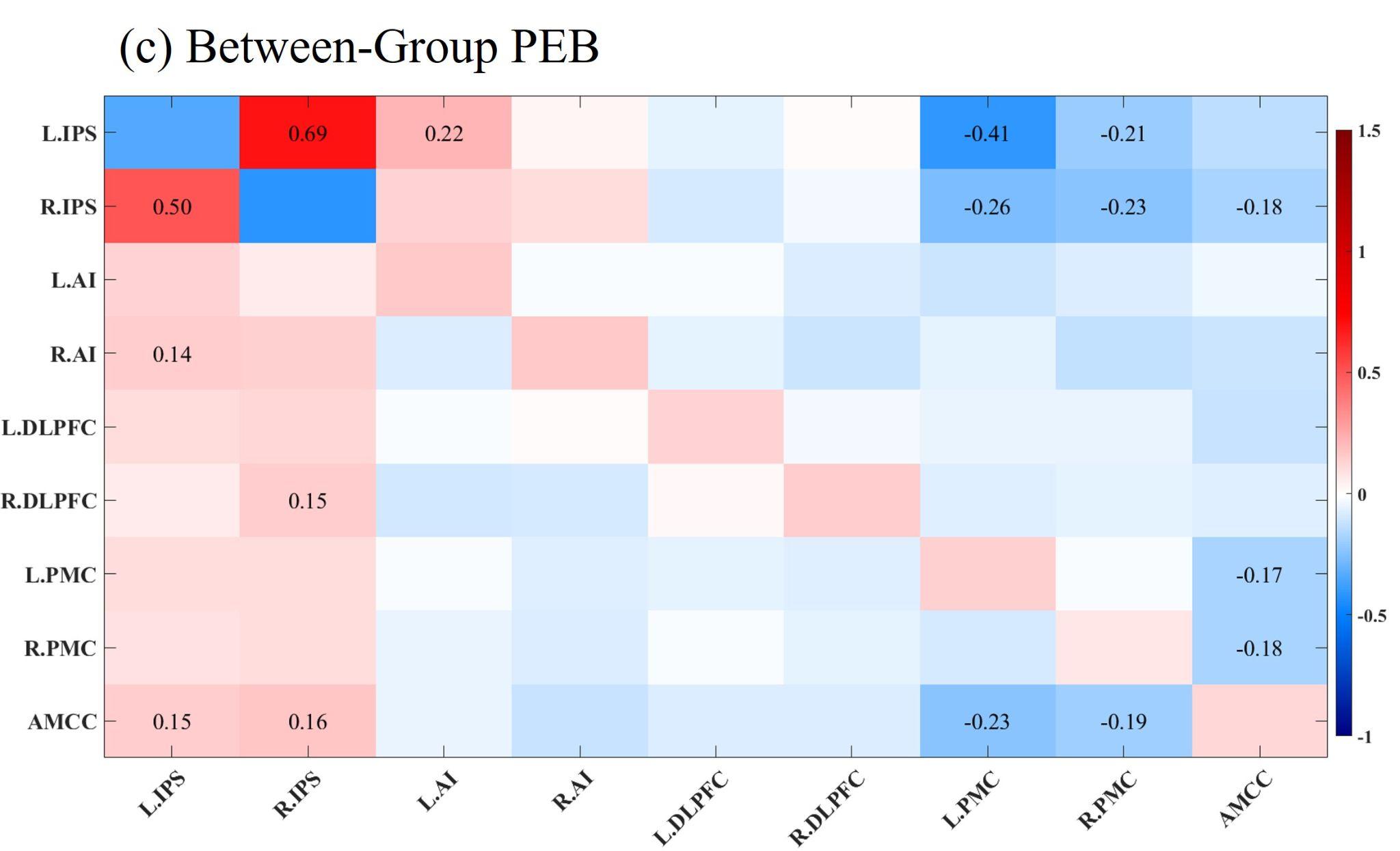** | (d)  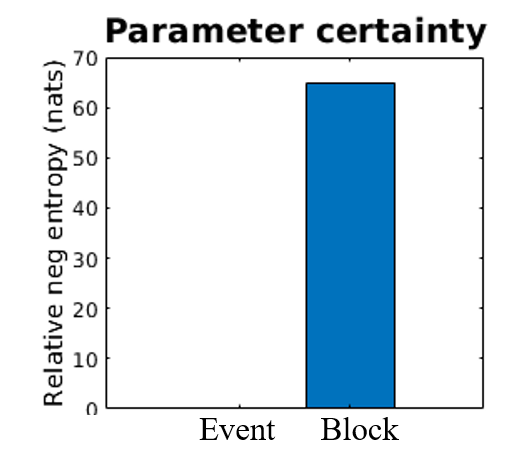 |

Fig. 12. Results of DCM calculations of the task-modulated EC (M-EC, matrix B) and its analysis for the case when the same BOLD signals extracted for the case of the block-based GLM of the uncorrected Anti+Pro contrast were supplied for modeling by the event-related and block-based DCM. (a) and (b) Connectivity patterns of the group-mean M-EC for event-related and block-based DCM designs as indicated in the titles of the plots. (c) and (d) The EC difference and parameter certainty between event-related and block-based designs. The strongly evident EC parameters (PP > 95%) are indicated by numbers in the matrix cells.

| 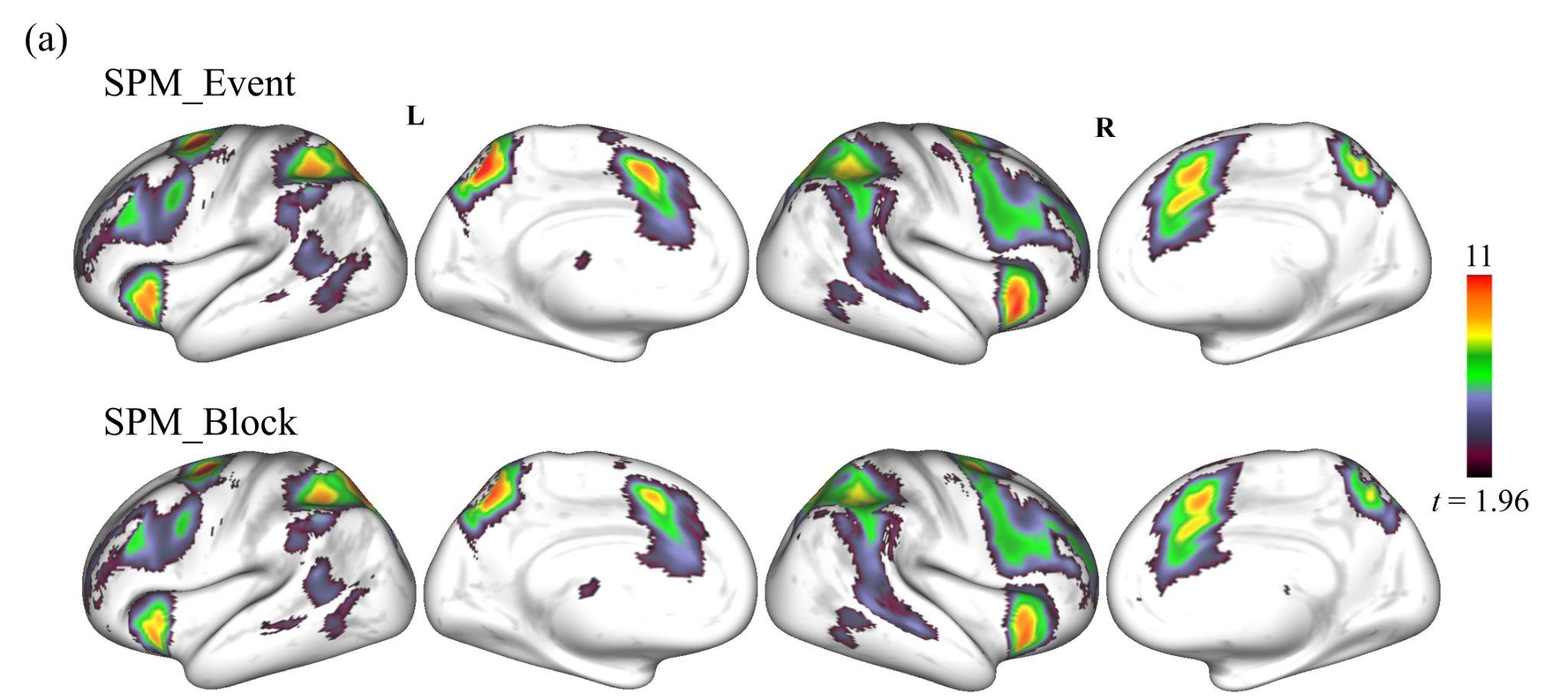 |
| --- |
| 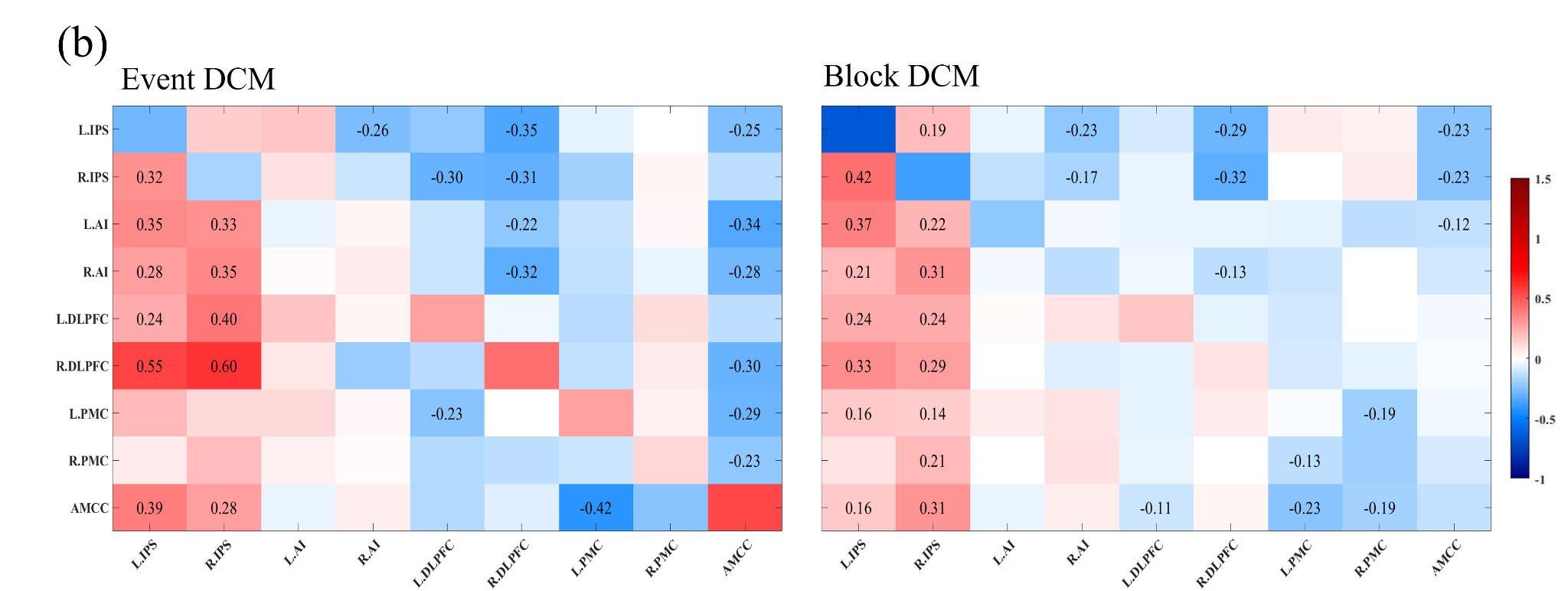 |
| 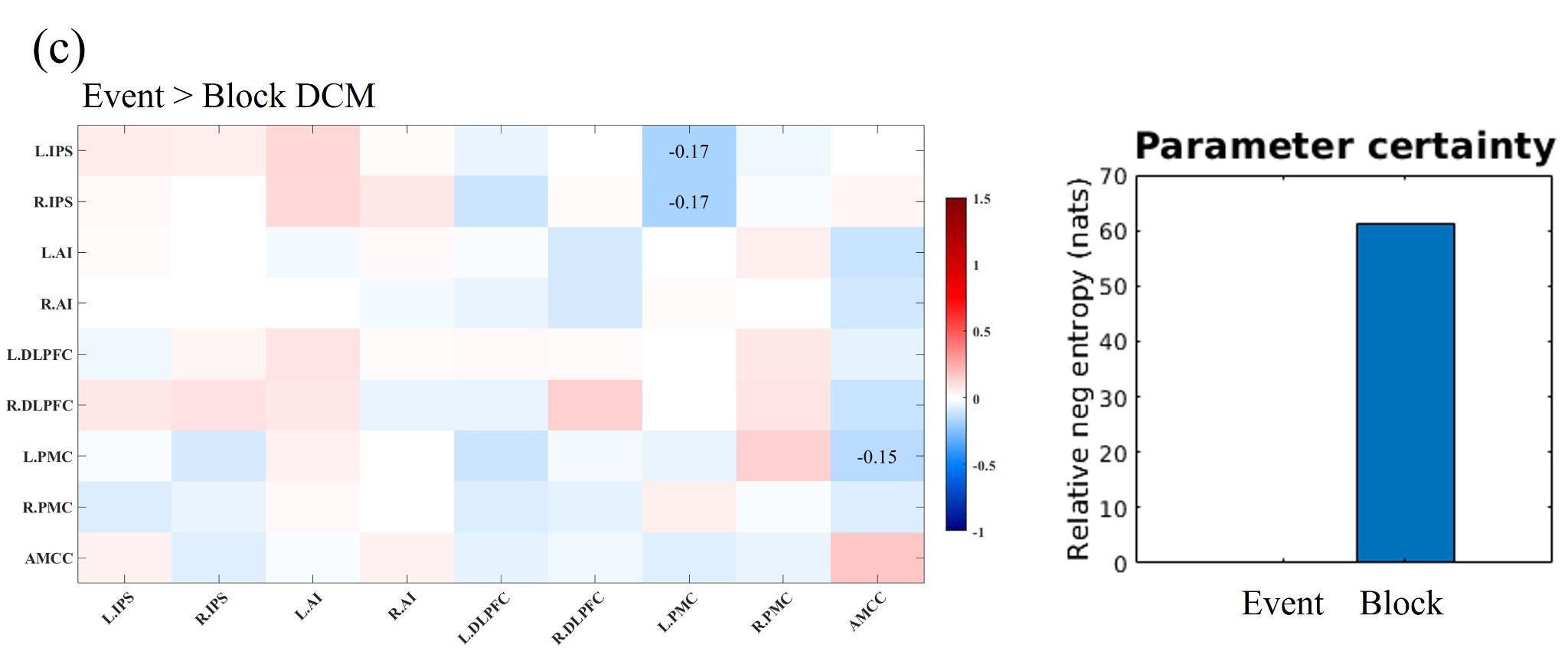 |

**Fig. 13** Results of second-level fMRI statistics and sequential DCM analyses of the task-modulated EC (M-EC, matrix B) between event-related and block-based uncorrected Anti+Pro cases using the processing of SPM. (a) Results of the second-level fMRI analysis with different general linear model (GLM) designs, and were corrected by *p*_TFCE_ < 0.05. (b) Connectivity patterns of the group-mean M-EC for event-related and block-based DCM designs as indicated in the titles of the plots. (c) The EC difference and parameter certainty between event-related and block-based designs. The strongly evident EC parameters (PP > 95%) are indicated by numbers in the matrix cells.

## Supplementary Methods

## SPM-based pipeline

Here we verified whether and how the reported results and derived conclusions can be influenced if different pipelines for the processing and analysis of task-evoked fMRI data would be used. We therefore repeated the investigation workflow and conducted the data processing by SPM pipeline, and then compared the results of the task-fMRI data processing and analyses as well as DCM-calculated task-evoked EC for event-related (AllTrials) and block-based designs of the general linear model (GLM) and DCM for the uncorrected Anti+Pro contrast. The SPM processing used spatially normalized and smoothed functional images obtained as described in the Methods, where all data processing and analysis of the task-fMRI data and DCM calculations were solely performed by SPM and included the following steps:

1. High-pass temporal filtering (128 s) was performed, and 27 regressors were convolved with hemodynamic response function during the first-level fMRI analysis by event-related and block-based GLM. Activation contrasts of Pro, Anti, Anti+Pro, and Anti-Pro were estimated.
2. A group-based activation as given by the Anti-Pro contrast was calculated and corrected by *p* < 0.05 with a family-wise error (FWE) rate (Supplementary Fig. S13a). The group-level peak coordinates (local maxima) were generated as SRC network nodes (Supplementary Table S11).
3. Individual peak coordinates generation and BOLD time series extraction were realized by the SPM volume-of-interest function that searched for the global maximal peak inside the spheres (radius = 10 mm) centered at the group-level peak coordinates, dilated the found individual peak of each regions of interest (ROI) into a sphere with a radius = 4 mm, employed the Anti+Pro contrast (p < 0.05 uncorrected) to remove insignificant voxels, and extracted the first eigenvariate as time series for DCM estimation.
4. Individual DCM estimation, calculation and checking of the explained variance (EV), evaluation of the group-mean EC by PEB, between-group PEB analysis, and Bayesian-data comparison (BDC) were the same as performed in the main text (see Methods).

Because of different data processing of the task-evoked fMRI, we observed some quantitative differences in results for the above SPM pipeline.

1. For the second-level fMRI analysis, we observed similar activation patterns between our analysis from the main text and the SPM pipeline (compare Fig. 2 and Supplementary Fig. S13a). For the later pipeline the Dice coefficient between the activation maps of the event-related and block-based GLM is 0.94 as before (Supplementary Table S3), and the mean absolute difference between peak coordinates is 0.3 mm vs. 0.7 mm as before (Supplementary Tables S1 and S11). There are small differences of peak coordinates between the pipelines of 1.6 mm and and 2.1 mm for the AllTrials and Block cases, respectively. The SPM pipeline in average led to somewhat smaller t-values of the activation peaks of 10.0 vs. 11.0 for the AllTrials and 9.3 vs. 10.4 for Blocks, which might be indicative for statistical quality of the modeled second-level activation maps (Supplementary Tables S1 and S11).
2. The same number of subjects (216) were qualified for the signal extraction and DCM analysis for the event-related case, while only 173 subjects (vs. 212) were approved by the SPM pipeline for the block-based design. We also observed differences in the modeling quality of DCM, where the pipeline from the main text offered a higher explained variance (EV) of DCM than the SPM pipeline of 24.6% vs. 19.1% and 22.9% vs.17.9% for the AllTrials and Block cases, respectively (Supplementary Tables S4 and S12).
3. Based on the group-mean PEB analysis, we observed fewer strongly evident M-EC edges passing the threshold of the posterior probability (PP) > 95% for the SPM pipeline of 25 vs. 39 (-36%) for AllTrials and 28 vs. 32 (-13%) for Blocks (Table 3 and Supplementary Fig. S13b). At the same time, the average absolute connectivity intensity was also reduced for the SPM pipeline for 0.33 vs. 0.56 (-41%) for AllTrials and 0.23 vs 0.26 (-12%) for Blocks (Supplementary Table S10 and Fig. S13b). Nevertheless, also for the SPM pipeline we still observed large differences in the group-mean connectivity patterns between event-related and block-based GLM and DCM designs (Supplementary Fig. S13b) as well as a larger connectivity intensity for the former design, but no pronounced difference in the number of strongly evident M-EC edges.
4. The between-group PEB analysis still found differences in M-EC (All-Trials > Block), but only in 3 EC connections as compared to 14 edges reported for the pipeline used in the main text (Fig. 5 and Supplementary Fig. S13c).
5. The BDC analysis consistently demonstrated higher parameter certainty (Block > AllTrials GLM designs) also for the SPM pipeline (61 nats, Supplementary Fig. S13c) comparable to our results presented in the main text (64 nats, Fig. 7).

In summary, application of the SPM pipeline largely confirmed our main conclusions, where we can still observe that event-related and block-based GLM designs showed differences in EC patterns in spite of the fact that the quality of the processed and analyzed data was affected. We in particular observed lower statistics of the second-level analysis, a smaller subset of participants qualified for the DCM analysis, a smaller fraction of the BOLD variance explained by DCM, and a strong reduction of the M-EC density and intensity with PP > 95%, especially, for the event-related GLM and DCM designs.

## Supplementary Tables

| **Table 1.** The MNI peak coordinates (x, y, z) of the local maxima of *t*-values of the second-level analysis of the Anti-Pro contrast without GSR. | | | | | | | | | | | | |
| --- | --- | --- | --- | --- | --- | --- | --- | --- | --- | --- | --- | --- |
| Peak | **All-Trials** | | | | **S-Trials** | | | | **Blocks** | | | |
|  | *x* | *y* | *z* | ***t*** | *x* | *y* | *z* | ***t*** | *x* | *y* | *z* | ***t*** |
| **LDLPFC** | -40 | 22 | 28 | **7.3** | -40 | 22 | 28 | **6.9** | -40 | 22 | 28 | **7.0** |
| **RDLPFC** | 36 | 30 | 28 | **7.1** | 36 | 30 | 28 | **6.9** | 36 | 26 | 24 | **6.2** |
| **LPMC** | -24 | -8 | 48 | **16.2** | -24 | -8 | 48 | **16.5** | -24 | -8 | 48 | **15.3** |
| **RPMC** | 24 | -6 | 50 | **11.8** | 24 | -8 | 48 | **12.2** | 24 | -6 | 50 | **11.0** |
| **LIPS** | -34 | -46 | 38 | **10.8** | -34 | -46 | 38 | **10.7** | -34 | -46 | 38 | **10.7** |
| **RIPS** | 36 | -44 | 40 | **10.3** | 36 | -44 | 40 | **10.6** | 36 | -44 | 40 | **9.9** |
| **LAI** | -32 | 18 | -10 | **11.8** | -32 | 18 | -10 | **10.6** | -32 | 18 | -10 | **11.4** |
| **RAI** | 30 | 20 | -4 | **13.1** | 30 | 20 | -4 | **12.5** | 30 | 20 | -4 | **12.1** |
| **AMCC** | 0 | 8 | 48 | **10.9** | -2 | 6 | 48 | **10.5** | 0 | 8 | 48 | **10.3** |

The corresponding local maximal *t*-values are also indicated (in boldface) of all SRC network nodes (first column), considered GLM designs (first row), and the case without GSR. SRC, stimulus-response compatibility; GLM, general linear model; GSR, global signal regression; All-/S-Trials, experimental designs with all trials or only successful trials; Blocks, experimental designs modeled by blocks; L/R, left/right; DLPFC, dorsolateral prefrontal cortex; PMC, premotor cortex; IPS, intraparietal sulcus; AI, anterior insula; AMCC, anterior midcingulate cortex.

| **Table 2.** Sample sizes for different conditions of the data processing without GSR | | | | | | |
| --- | --- | --- | --- | --- | --- | --- |
|  | **All-Trials** | | **S-Trials** | | **Blocks** | |
|  | Corrected | Uncorrected | Corrected | Uncorrected | Corrected | Uncorrected |
| **Anti** | 163/160 | 215/209 | 153/151 | 211/207 | 173/168 | 222/213 |
| **Anti+Pro** | 178/176 | 221/216 | 170/170 | 213/208 | 185/182 | 218/212 |

The two subject numbers given in each table cell correspond to the subject samples qualified for BOLD signal extraction for SRC network nodes of individual subjects/explained variance criterion of dynamic causal modeling, see Sec. 2.6 / Sec. 2.7 for details. Used notations: GSR, global signal regression; All-/S-Trials, experimental designs with all/successful trials; Blocks, experimental designs modeled by blocks; Anti, incompatible contrast; Anti+Pro, incompatible+compatible contrast.

| **Table 3.** Dice’s value across different GLM designs with GSR | | | |
| --- | --- | --- | --- |
| Dice’s D | All-Trials | S-Trials | Blocks |
| All-Trials | 1 | 0.94 | 0.94 |
| S-Trials | 0.94 | 1 | 0.93 |
| Block | 0.94 | 0.93 | 1 |

Dice’s D is calculated by the following equation: D=2C/(A+B). C denotes overlapped voxels; A and B denote voxels in the condition of A and B. All-Trials, general linear model (GLM) design with all stimuli and responses; S-Trials, GLM design with successfully-responded stimuli and responses; Blocks, GLM design coded by block onset and duration information. GSR, global signal regression.

| **Table 4.** Averaged explained variance (EV) and its standard deviation estimated for all conditions. | | | | | | |  |
| --- | --- | --- | --- | --- | --- | --- | --- |
|  | **All-Trials** | | **S-Trials** | | **Blocks** | | |
|  | Corrected | Uncorrected | Corrected | Uncorrected | Corrected | Uncorrected | |
| Anti^1^ | 23.9±7.1 | 22.6±7 | 23.1±6.3 | 21.6±6.4 | 23.7±7.6 | 22.6±7.5 | |
| Anti+Pro^1^ | 25.4±6.9 | 24.6±7 | 24.4±6.6 | 23.6±6.6 | 23.6±7 | 22.9±7 | |
| Anti^2^ | 24±7.7 | 23±7.4 | 23.4±7.1 | 22±6.9 | 23.7±7.8 | 22.8±7.9 | |
| Anti+Pro^2^ | 25.1±7.5 | 24.4±7.6 | 24.5±7.4 | 23.8±7.3 | 23.6±7.7 | 23.3±7.6 | |

Note: ^1^ indicates the conditions with global signal regression (GSR), while ^2^ indicates the conditions without GSR. All-/S-Trials, experimental designs with all/successful trials; Blocks, experimental designs modeled by blocks; Anti, incompatible contrast; Anti+Pro, incompatible+compatible contrast.

| **Table 5.** Numbers of the group-level EC edges of modulatory (matrix B) EC (PP > 95%) within the SRC network without GSR. | | | | | | |
| --- | --- | --- | --- | --- | --- | --- |
|  | **All-Trials** | | **S-Trials** | | **Blocks** | |
|  | Corrected | Uncorrected | Corrected | Uncorrected | Corrected | Uncorrected |
| Anti | 34 | 40 | 32 | 43 | 12 | 18 |
| Anti+Pro | 36 | 40 | 37 | 40 | 33 | 35 |

All modulatory effective connectivity (EC) connections were exceeding 95% of the posterior probability (excluding self-connections) calculated by Parametric Empirical Bayes (PEB) for the considered conditions of the data processing without global signal regression (GSR). All-/S-Trials, experimental designs with all/successful trials; Blocks, experimental designs modeled by blocks; Anti, incompatible contrast; Anti+Pro, incompatible+compatible contrast.

| **Table 6.** Relative differences in parameter certainty between GLM designs estimated from Bayesian Data Comparison (BDC) | | | | | | |
| --- | --- | --- | --- | --- | --- | --- |
| GSR | Block > All-Trials | | Block > S-Trials | | All-Trials > S-Trials | |
|  | Anti | Anti+Pro | Anti | Anti+Pro | Anti | Anti+Pro |
| Corrected | 58.1 | 64.2 | 62.9 | 62.4 | 2.3 | -1.1 |
| Uncorrected | 66.5 | 64.0 | 66.7 | 63.3 | 0.5 | -0.9 |

Note: the number in each element indicates a relative difference in parameter certainty estimated from the BDC analysis between conditions of different general linear model (GLM) designs. Used notations are the same as in Table 5. The relative value was extracted between two groups (same size) using Bayesian data comparison (spm_dcm_bdc.m), and represents the relative levels of parameter certainty (nats) of the estimated model parameter. A difference between two data sets in the range between 1.1 and 3 nats (natural units) and between 3 and 5 nats can be considered as “positive evidence” and “strong evidence”, respectively. The higher value indicates higher parameter certainty in one condition relative to the other one. For example, Block-based designs showed very strong evidence in parameter certainty compared to event-related designs.

| **Table 7.** Relative differences in parameter certainty between activation contrasts (Anti > Anti+Pro) estimated from Bayesian Data Comparison (BDC) | | | | | | |
| --- | --- | --- | --- | --- | --- | --- |
| Activation contrast | AllTrials | | STrials | | Block | |
|  | Corrected | Uncorrected | Corrected | Uncorrected | Corrected | Uncorrected |
| Value | 9.7 | 7.9 | 7.2 | 7.0 | 10.4 | 11.1 |

Note: the number in each element indicates a relative difference in parameter certainty between activation contrasts (Anti > AntiPro) estimated from the BDC analysis. Used notations are the same as in Table 6.

| **Table 8.** Relative differences in parameter certainty between conditions of GSR (with GSR > without GSR) estimated from Bayesian Data Comparison (BDC) | | | | | | |
| --- | --- | --- | --- | --- | --- | --- |
| GSR | All-Trials | | S-Trials | | Block | |
|  | Anti | Anti+Pro | Anti | Anti+Pro | Anti | Anti+Pro |
| Corrected | -0.3 | -1.9 | -0.7 | -1.4 | 0.4 | 1.5 |
| Uncorrected | 0.4 | -2.2 | -0.3 | -2.6 | 0.1 | 1.6 |

Note: the number in each element indicates a relative difference in parameter certainty between conditions of with GSR and without GSR from the BDC analysis. Used notations are the same as in Table 5.

| **Table 9.** Relative differences in parameter certainty (Corrected > Uncorrected) | | | | | | |
| --- | --- | --- | --- | --- | --- | --- |
| Significance  thresholding | All-Trials | | S-Trials | | Block | |
|  | Anti | Anti+Pro | Anti | Anti+Pro | Anti | Anti+Pro |
| Value | 1.1 | 0.4 | 0.03 | 0.7 | -0.2 | -0.2 |

Note: the number in each element indicates a relative difference in parameter certainty between conditions of corrected and uncorrected from the BDC analysis. Used notations are the same as in Table 5.

| **Table 10.** Averaged absolute intensity of task-evoked modulatory EC (PP > 95%) of all conditions | | | | | | |
| --- | --- | --- | --- | --- | --- | --- |
|  | **All-Trials** | | **S-Trials** | | **Blocks** | |
|  | Corrected | Uncorrected | Corrected | Uncorrected | Corrected | Uncorrected |
| Anti^1^ | 0.48 | 0.46 | 0.49 | 0.46 | 0.34 | 0.33 |
| Anti+Pro^1^ | 0.55 | 0.54 | 0.56 | 0.53 | 0.27 | 0.26 |
| Anti^2^ | 0.47 | 0.45 | 0.49 | 0.44 | 0.33 | 0.30 |
| Anti+Pro^2^ | 0.52 | 0.5 | 0.55 | 0.52 | 0.27 | 0.26 |

Note: ^1^ indicates the conditions with GSR, while ^2^ indicates the conditions without GSR. Each element in this table indicates the averaged absolute value of effective connectivity (EC) for each condition. All-Trials, general linear model (GLM) design with all stimuli and responses; S-Trials, GLM design with successfully-responded stimuli and responses; Blocks, GLM design coded by block onset and duration information. Corrected, the condition of corrected significance thresholding; Uncorrected, the condition of uncorrected significance thresholding.

| Table 11. MNI peak coordinates (x, y, z) of the local maxima of *t*-values based on the second-level fMRI statistics of the Anti-Pro contrast (from SPM processing) | | | | | | | | |
| --- | --- | --- | --- | --- | --- | --- | --- | --- |
| Peak | **All-Trials** | | | | **Blocks** | | | |
|  | *x* | *y* | *z* | ***t*** | *x* | *y* | *z* | ***t*** |
| **LDLPFC** | -40 | 28 | 28 | 6.6 | -40 | 28 | 28 | 6.4 |
| **RDLPFC** | 36 | 30 | 26 | 6.8 | 38 | 32 | 26 | 6.8 |
| **LPMC** | -24 | -8 | 48 | 13.9 | -24 | -8 | 48 | 12.5 |
| **RPMC** | 24 | -8 | 48 | 10.8 | 24 | -8 | 48 | 9.7 |
| **LIPS** | -32 | -46 | 38 | 9.6 | -32 | -46 | 38 | 9.8 |
| **RIPS** | 38 | -48 | 42 | 8.9 | 38 | -48 | 42 | 8.4 |
| **LAI** | -30 | 18 | 0 | 10.8 | -30 | 18 | 0 | 9.8 |
| **RAI** | 30 | 18 | 0 | 12.3 | 30 | 18 | 0 | 11.1 |
| **AMCC** | -6 | 4 | 46 | 10.7 | -4 | 6 | 46 | 9.5 |

The corresponding local maximal *t*-values are also indicated (in boldface) of all SRC network nodes (first column), considered GLM designs (first row), and the case based on the SPM processing. SRC, stimulus-response compatibility; GLM, general linear model; All-/S-Trials, experimental designs with all trials or only successful trials; Blocks, experimental designs modeled by blocks; L/R, left/right; DLPFC, dorsolateral prefrontal cortex; PMC, premotor cortex; IPS, intraparietal sulcus; AI, anterior insula; AMCC, anterior midcingulate cortex.

Table 12. Summary of subject cohort size qualified for signal extraction and DCM analyses, variance of BOLD signals explained by DCM, and connectivity intensity for the SPM processing

| GLM designs | Subjects | Explained variance | Absolute intensity |
| --- | --- | --- | --- |
| All-Trials | 258/216 | 19.1±7.4 % | 0.33 |
| Blocks | 256/173 | 17.9±8.8 % | 0.23 |

The two subject numbers given in “Subjects” cells correspond to the subject samples qualified for BOLD signal extraction for SRC network nodes of individual subjects/explained variance criterion of DCM,. The two numbers given in “Explained variance” cells correspond to average and standard deviation of explained variance for event-related and block-based GLM cases. The absolute intensity was calculated by average absolute intensity value of evident EC (posterior probability > 95%).
